# Revealing the Combined Effect of Active Sites and Intra-Particle Diffusion on Adsorption Mechanism of Methylene Blue on Activated Red-Pulp Pomelo Peel Biochar

**DOI:** 10.3390/molecules28114426

**Published:** 2023-05-29

**Authors:** Fang Wei, Shenglong Jin, Chunyi Yao, Tianhao Wang, Shengpu Zhu, Yabiao Ma, Heng Qiao, Linxi Shan, Rencong Wang, Xiaoxue Lian, Xiaoqiang Tong, Yan Li, Qiang Zhao, Weiguo Song

**Affiliations:** 1 College of Science, Civil Aviation University of China (CAUC), Tianjin 300300, China; f_wei@cauc.edu.cn (F.W.); 18893755317@163.com (S.Z.); w386580571@gmail.com (R.W.); lianxiaoxues@163.com (X.L.); txq2009@126.com (X.T.); liyan01898@163.com (Y.L.); 2 Laboratory of Molecular Nanostructure and Nanotechnology, Institute of Chemistry, Chinese Academy of Sciences, Beijing 100190, China; wsong@iccas.ac.cn

**Keywords:** activated biochar, pyrolysis mechanisms, adsorption thermodynamics, kinetic models, mechanisms of adsorption dye

## Abstract

Phosphoric acid-activated biochar has been proven to be a promising adsorbent for pollutant removal in an aqueous solution. It is urgent to understand how surface adsorption and intra-particle diffusion synergistically contribute to the adsorption kinetic process of dyes. In this work, we prepared a series of PPC adsorbents (PPCs) from red-pulp pomelo peel under different pyrolysis temperatures (150–350 °C), which have a broad specific surface area range from 3.065 m^2^/g to 1274.577 m^2^/g. The active sites on the surface of PPCs have shown specific change laws of decreasing hydroxyl groups and increasing phosphate ester groups occurring as the pyrolysis temperature rises. Both reaction models (PFO and PSO models) and diffusion models (intra-particle diffusion models) have been applied to simulate the adsorption experimental data to verify the hypothesis deduced from the Elovich model. PPC-300 exhibits the highest adsorption capacity of MB (423 mg/g) under given conditions. Due to its large quantities of active sites on the external and internal surfaces (1274.577 m^2^/g), a fast adsorption equilibrium can be achieved within 60 min (with an initial MB concentration of 100 ppm). PPC-300 and PPC-350 also exhibit an intra-particle-diffusion-controlled adsorption kinetic process with a low initial MB concentration (100 ppm) or at the very beginning and final stage of adsorption with a high initial MB concentration (300 ppm) at 40 °C, considering that the diffusion is likely hindered by adsorbate molecules through internal pore channels at the middle stage of adsorption in these cases.

## 1. Introduction

Activated biochar has been widely investigated as a promising alternative to activated carbons (ACs) in terms of adsorption functionality [1]. As is well known, ACs have been generally produced from conventional fossil feedstocks in the past few decades, which exhibits disadvantages, such as unsustainability, high carbon footprint, complex production flows, high energy consumption, and low production yield. Activated biochar is a kind of carbonaceous solid material produced from a wide range of biomass feedstocks (e.g., agricultural waste, fruit peels, plant roots, or even domestic wastewater sludge) under less energy-intensive thermal conditions [2]. Benefiting from the wide range of these feedstocks and the great diversity of activation strategies, it brings more heterogeneity in biochar’s surface groups than that in ACs’ polycyclic aromatic networks. On the other hand, they have also shared commonalities in terms of high aromaticity in their carbonized phases and high porosity in their bulk structures. It is evident that the morphological structures (particle size, surface area), bulk properties (porosity and aromaticity), and surface properties (surface charge, polarity, chemical groups) are critical issues in activated biochar research for adsorption application [3].

In order to elucidate the probable adsorption mechanisms of organic compounds binding to activated biochar, the intermolecular interaction forces should be carefully discussed based on the molecular structure characteristics of adsorbents (activated biochar) and adsorbates (aqueous-phase organic pollutants such as organic dyes, phenols, fertilizers, pesticides, antibiotic drugs) [4,5,6]. In general, covalent bonding, coordinate bonding, and even H-bonding with strong electron transfer are common driving forces of chemisorption, whereas non-covalent intermolecular forces such as coulombic forces, π–interactions, dipole interactions, and hydrophobic interactions, are mainly driving forces of physisorption [7]. To be specific, it is hard to draw confident conclusions about the adsorption mechanism without enough experimental and simulated pieces of evidence. Additionally, the adsorption thermodynamic and kinetic studies are needed to achieve a reasonable comprehension of adsorption behavior when combined with other experimental approaches [8]. 

Pomelo peels are mainly composed of pectin, hemicellulose, cellulose, and lignin, which are ideal carbon sources for producing activated biochar [9]. According to our previous research, the phosphoric acid-activated pomelo peel chars (PPCs) have shown great adsorption affinity for cationic dyes in neutral and alkaline aqueous systems due to the coulombic attraction driving force, which is proved by the pH dependency of adsorption quantities for cationic dyes [10]. The oxygen-containing groups (phosphate groups, carboxylic groups, and hydroxyl groups) are considered probable major adsorption sites in PPCs’ structures. The pH is a crucial factor in converting coulombic forces from attraction to repulsion between adsorbents and adsorbates in experiments. However, we observed large adsorption quantities of cationic dyes excluding the contributions of coulombic force. PPC also shows a high adsorption capacity of MB under acidic conditions (coulombic repulsion force is dominant in this case), indicating the existence of different adsorption mechanisms except for coulombic force. 

To reveal its adsorption mechanisms, there are some critical issues that should be addressed: (1) Methylene blue (MB) has a thiazine conjugated structure that is favorable for the formation of π–π interactions, a quaternary ammonium group with a positive charge that is favorable for the formation of coulombic force, and a tertiary ammonium group with a lone pair of electrons that is favorable for the formation of H-bonding and n–π interactions. On the other hand, PPCs have high aromaticity of fused aromatic rings, which are the fundamental basis of forming electron-rich π-systems that are capable of being attractive to polar molecules and other π-systems. Nonetheless, we still have no comprehensive understanding of how these intermolecular interactions contribute to the adsorption kinetic process; (2) PPCs have a large portion of micropores (<2 nm), connecting to mesopores (2 nm to 50 nm), and opening to the outside surface. The steric hindrance effect occurs when the pore aperture is too narrow for the MB molecule’s diffusion to deeper sites on the internal surface. In this case, the adsorption is probably limited by the pore/surface (intra-particle) diffusion. However, we also have no evidence to understand at which stage of adsorption or in which condition it exhibits a diffusion-controlled adsorption process. In general, we are going to answer how surface adsorption and intra-particle diffusion synergistically contribute to the adsorption kinetic mechanism of MB on PPCs, which have not been further investigated in our previous work.

In this article, we prepared a series of PPCs from red-pulp pomelo peel under different pyrolysis temperatures (150–350 °C). The structural morphology, N_2_ adsorption-desorption isotherm, pore size distribution, surface chemistry, and aromaticity of the carbon framework of PPCs have been investigated. The adsorption isotherm models including the Langmuir model, Freundlich model, and Temkin model have been used to simulate the experimental data to evaluate the adsorption performance of MB on PPCs. Both reaction models (pseudo-first-order kinetic model and pseudo-second-order kinetic model) and intra-particle diffusion models (Weber-Morris model and Boyd model) have also been applied to simulate the adsorption experimental data in order to verify the hypothesis deduced from the Elovich model. In general, we consider all these factors to give probable adsorption mechanisms of MB on PPCs by this method.

## 2. Results

### 2.1. Morphology and Textural Properties of PP and PPCs

**Figure 1:**
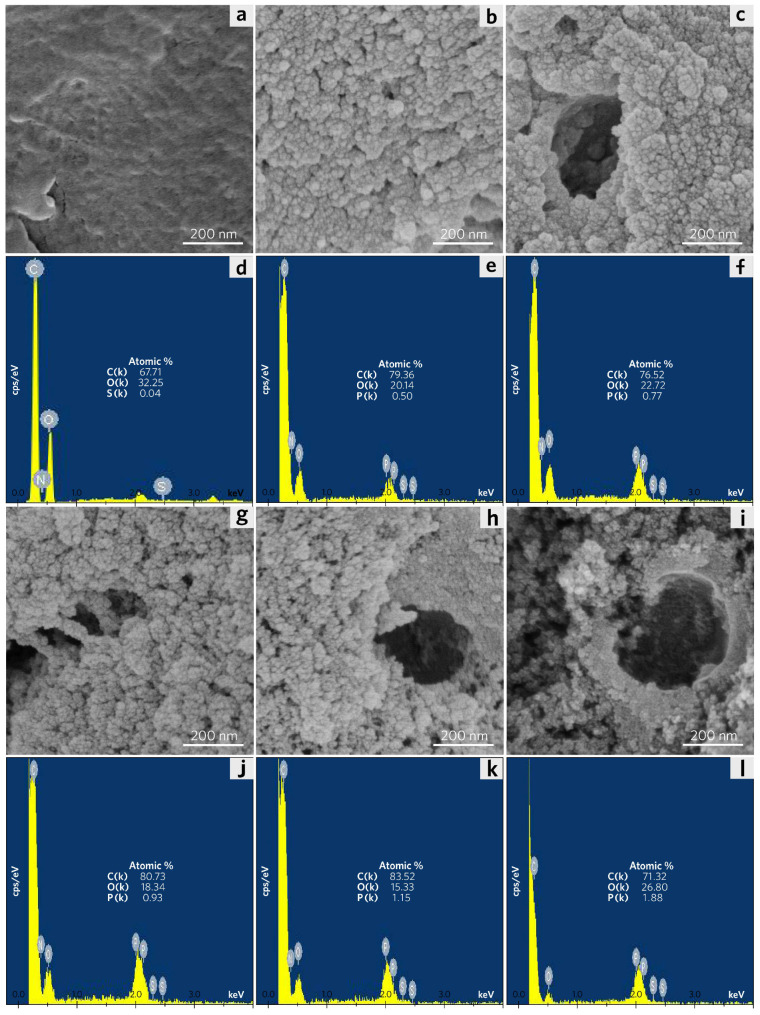
SEM images and EDS patterns with atomic ratio of C, O, and P element of (**a**,**d**) PP, (**b**,**e**) PPC-150, (**c**,**f**) PPC-200, (**g**,**j**) PPC-250, (**h**,**k**) PPC-300, (**i**,**l**) PPC-350.

The microscopic morphology and surface elemental analysis of PP and PPCs were identified by scanning electron microscopy (SEM) and energy dispersive spectrum (EDS) as shown in Figure 1. 

Figure 1a reveals that PP has a soft surface morphology with abundant smooth wrinkles. As shown in Figure 1d, according to the EDS analysis of PP, it shows that there are elements including abundant C, and O with a C/O atomic ratio of 2.10. According to the SEM images (see Figure 1b,c,g–i), as the pyrolysis temperature rises, the irregular and heterogeneous surface increases in PPCs. This contributes to PPCs’ high specific surface areas and large total pore volumes. Especially, PPC-250, PPC-300, and PPC-350 have shown enhanced sponge-like structures, which significantly differ from that of the other PPCs (PPC-150 and PPC-200). Therefore, it can be concluded that the morphology of the PPCs is strongly dependent on the pyrolysis temperature. The corresponding C/O atomic ratios of PPCs are ranked as follows: PPC-300 (5.45, in Figure 1k) > PPC-250 (4.40, in Figure 1j) > PPC-150 (3.94, in Figure 1e), PPC-200 (3.36, in Figure 1f) > PPC-350 (2.66, in Figure 1l). The increase in the C/O ratio represents the decomposition of cellulose and hemicellulose in the synthesis of PPCs above 250 °C. Whereas, the decrease in the C/O ratio indicates the decomposition of lignin at high-temperature thermal treatment (above 350 °C). This will be discussed in the thermal analysis section.

**Figure 2:**
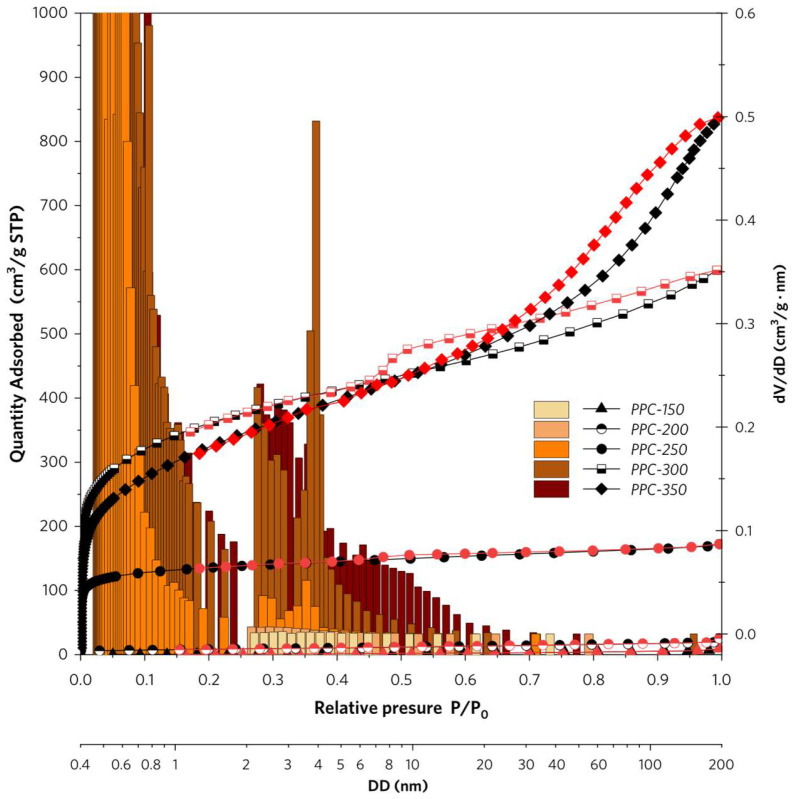
N_2_ adsorption-desorption isotherms and pore size distribution bar charts of PPCs.

The BET-specific surface area of PP is 0.5503 m^2^/g due to its smooth and nonporous structure. As shown in Figure 2 and Table 1, PPC-300 exhibits the highest specific surface area being 1274.577 m^2^/g, whereas PPC-150 has the lowest specific surface area being 3.065 m^2^/g. A significant increase in specific surface area is attributed to the generation of abundant micropores and mesopores in PPCs. Generally, porous structures are generated through volatilization of small molecules (H_2_O and organic matter of low molecular weight) and gasification of partial carbon atoms (CO_2_, CO, CH_4_ produced in oxidation reaction) in thermal treatment of PPC precursor. As the temperature rises, micropores are generated prior to the formation of mesopores in PPCs. PPC-300 possesses the most total micropore volume (0.5105 cm^3^/g). However, PPC-350 has a decrease both in the total micropore volume and the volume proportion of micropores, which indicates a new stage of pyrolysis occurs above 300 °C. This will be fully discussed in the thermal analysis section of PP and PPCs as below.

### 2.2. Thermal Analysis of PP and PPCs

**Figure 3:**
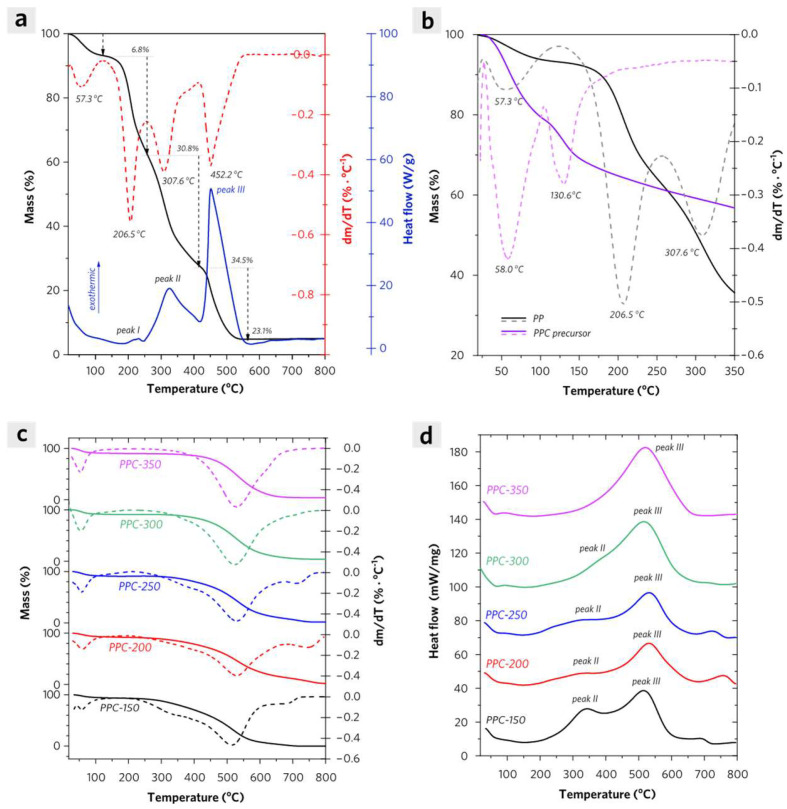
(**a**) TGA, DTA, and DSC curves of PP in air at a heating rate of 5 °C/min within a temperature range from room temperature to 800 °C, (**b**) TGA and DTA curves of PP and PPC precursor in air at a heating rate of 5 °C/min within temperature range from room temperature to 350 °C. (**c**) TGA, DTA, and (**d**) DSC curves of PPCs in air at a heating rate of 5 °C/min within a temperature range from room temperature to 800 °C.

In order to understand the synthesis of PPCs, thermal analysis experiments have been performed as shown in Figure 3. The TGA and DSC analysis of PP in the air has revealed its major compositions including hemicellulose, cellulose, and lignin. According to Figure 3a, the mass loss curve of PP has a continuous declination as the temperature rises. Its differential thermal analysis (DTA) curve shows four individual peaks which are mainly successively attributed to the loss of adsorbed water with almost 6.8% of total mass (57.3 °C), the decomposition of hemicellulose with almost 30.8% of total mass (206.5 °C), the decomposition of cellulose with almost 34.5% of total mass (307.6 °C), and the decomposition of lignin with almost 23.1% of total mass (452.2 °C). Meanwhile, the synchronized DSC curve has shown multiple exothermic peaks assigned to the oxidation process of hemicellulose (peak I), cellulose (peak II), and lignin (peak III) of PP. 

In Figure 3b, there are great differences in mass loss curves between the pyrolysis behavior of PP and PPC precursor (PP was impregnated with phosphoric acid for 24 h). Although, the loss of adsorbed water contributes to the mass loss of PP below 100 °C, and PPC precursor exhibits a similar mass loss procedure induced by water evaporation in the system. It is evident that a significant mass loss procedure occurs in a temperature range from 104.7 °C to 350 °C for PPC precursor, which is assigned to the formation of polyphosphoric acids and oxidative decomposition of hydrocarbons. The decomposition of PP begins at nearly 128.9 °C, in which exists the pyrolysis of hemicellulose with a maximum mass loss rate of 0.413 wt%/°C at 206.5 °C and the pyrolysis of cellulose with a maximum mass loss rate of 0.672 wt%/°C at 307.6 °C. The decomposition of PPC precursor has been suppressed above 200 °C with a very low mass loss rate of less than 0.067 wt%/°C. After the loss of adsorbed water, the condensed phosphoric acid becomes a mixture of phosphoric acids and polyphosphoric acids, including species such as H_3_PO_4_, H_4_P_2_O_7_, H_5_P_3_O_10_ in higher proportion, and H_n+2_P_n_O_3n+1_ (*n* > 4) in lower proportion [11]. In PPCs, a more highly cross-linked structure is developed by the esterification reaction between polyphosphoric acids and hydroxyl groups in PPC precursors, which results in the deceleration of mass loss caused by the oxidation and decomposition of hemicellulose and cellulose in synthesis. In conclusion, PPC-300 possesses the largest amounts of micropores, of which formation is due to the decomposition of all hemicellulose and part cellulose during calcination at 300 °C in air. Whereas PPC-350 possesses the largest amounts of mesopores which are attributed to the collapse of micropores that subsequently changes to mesopores at higher calcination temperature (>300 °C).

The TGA and DTA curves of PPCs are shown in Figure 3c. Due to the formation of the cross-linked structures, the decomposition temperature of hemicellulose, cellulose, and lignin slightly increases in PPCs as compared with PP. Except for the water loss process at a similar specific temperature range (50–80 °C), PPC keeps almost zero mass loss under its thermal treatment temperature, but it shows an obvious mass loss above this temperature in air. For PPC-150, PPC-200, or PPC-250, there is an obvious shoulder peak (at nearly 300 °C) adjacent to the broad main differential peak (at nearly 500 °C) in each of their DTA curves. Whereas this shoulder peak almost disappears in the DTA curves of PPC-300 and PPC-350, indicating cellulose has been almost destroyed via pyrolysis above 300 °C. This is in accordance with the above-discussed results. In Figure 3d, there are also both exothermic peaks II assigned to the decomposition of cellulose in PPCs and exothermic peaks III assigned to the decomposition of lignin in the DSC curves of PPCs except that there exists a single peak III in DSC curves of PPC-350, which indicates intensively exothermic decomposition of biomass under calcination in air.

In general, small molecules (volatiles with major groups such as C=O, C−O−C, etc.) are mainly released under pyrolysis below 200 °C, decreasing the C/O atomic ratio of PPC-200 as compared with that of PPC-150. The micropores are mainly generated from the decomposition of hemicellulose (150−250 °C). The mesopores are then formed by the decomposition of cellulose (250−350 °C), leading to the gasification of partial carbon atoms and the release of CO_2_ and CO, which increases the C/O atomic ratio of PPCs [12]. For PPC-350, some proportion of the micropores start to collapse above 300 °C. The C/O atomic ratio of PPC-350 decreases as compared with that of PPC-300, which is mainly attributed to the initial decomposition of lignin with a minor release of CH_4_ at higher temperatures than 300 °C [13].

### 2.3. Surface Chemistry and Characteristics of PPCs

**Figure 4:**
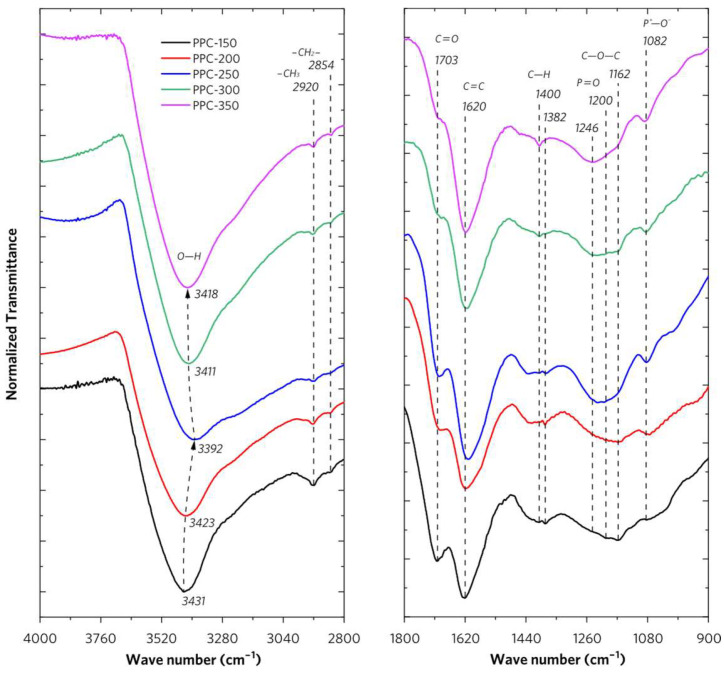
FTIR spectra of PPCs.

The major functional groups of PP are depicted in Figure S2, which includes the IR adsorption peaks of O–H at ~3420 cm^−1^ (ν) and 1330~1400 cm^−1^ (δ), C–H at 2800~3000 cm^−1^ (ν) and 1350~1450 cm^−1^ (δ), ester C=O at 1730~1750 cm^−1^ (ν), aromatic C=C at 1500~1650 cm^−1^ (δ), alcohol C–O at 1050~1100 cm^−1^ (ν), and ether C–O–C at 1150~1250 cm^−1^ (ν). The FT-IR spectra of PPCs are shown in Figure 4. The broad absorption bands located around 3390–3430 cm^−1^ are assigned to the O–H stretching vibrations of hydroxyl groups and adsorbed water in PPCs. PPC-250 has shown the largest redshift of O–H stretching vibrations absorption (3392 cm^−1^), indicating the enhanced hydrogen bond interactions of associative hydroxyl in PPC-250. For PPCs, the higher the thermal treatment temperature rises, the less the number of hydroxyl reserves in their complex structures. The absorption bands observed at 2920 cm^−1^ (ν_C–H_), 2854 cm^−1^ (ν_C–H_), 1434 cm^−1^ (δ_C–H_), and 1382 cm^−1^ (δ_C–H_) can be attributed to C–H stretching and bending vibrations of methyl (-CH_3_), methylene (-CH_2_-), and methoxy groups (-OCH_3_) in PPCs. The stretching vibration of the C=O absorption bands has a redshift changing from 1743 cm^−1^ (PP) to 1703 cm^−1^ (PPCs), which reveals the loss of ester carbonyl groups (-COOCH_3_) in pectin and the formation of aromatic carboxylic acid in the structure of lignin in PPCs. It is concluded that phosphate acid plays a crucial role in activating the hydrolysis of the ester. The bands at 1626 cm^−1^ can be assigned to the C=C stretching vibration of the aromatic ring structure in PP (lignin). As for PPCs, this band has shifted to 1620 cm^−1^, which indicates the enhanced degree of aromatic ring conjugation in PPCs. 

As shown in Figure S2, PP exhibits a distinct IR absorption spectrum as compared with PPCs. The band at 1331 cm^−1^ is evident for O–H bending vibrations of alcoholic hydroxyl groups. The bands at 1250 cm^−1^ and 1150 cm^−1^ represent the C−O−C stretching vibrations of the aromatic ether of lignin and aliphatic ether of cellulose or hemicellulose, respectively. The bands at 1104 cm^−1^ and 1055 cm^−1^ are assigned to the C−O bending mode of secondary and primary alcohol for cellulose and lignin. In PPCs IR spectra, the intensity ratio of absorption bands at 1246 cm^−1^ and 1162 cm^−1^ distinctly changed. The bands at 1162 cm^−1^, due to the C−O−C stretching vibrations of the aliphatic ether of cellulose or hemicellulose in PPCs, tend to disappear as the thermal treatment temperature rises from 150 °C to 350 °C. The bands at 1246 cm^−1^ which are assigned to the C−O−C stretching vibrations of the aromatic ether of lignin in PPCs, are basically unchanged. These results well matched with the conclusions based on thermal analysis.

For PPCs, the absorption bands at 1200 cm^−1^ are primarily assigned to the stretching mode of P=O, to O–C stretching vibrations in P–O–C (aromatic) linkage, and to P(=O)OH. The shoulders at 1082 cm^−1^ may be assigned to ionized linkage P^+^–O^–^ in phosphate esters [14,15,16]. These enhanced absorption bands also represent the increase of bonded phosphate groups in complex structures of PPCs.

**Figure 5:**
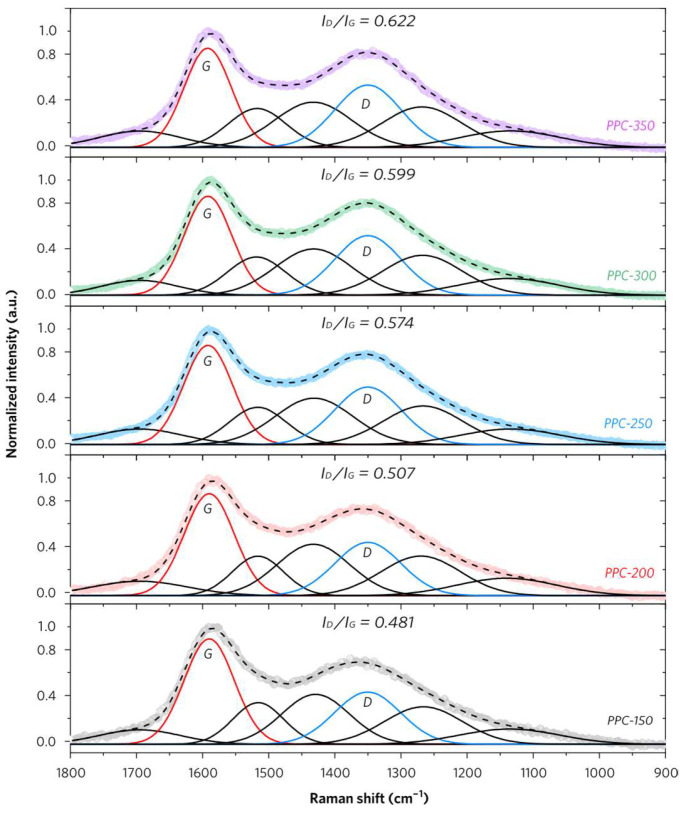
Raman spectra of PPCs and their deconvoluted peaks.

The Raman spectra of PPCs provide insights into the polarizable vibrations of C=C in aromatics and molecular backbones of carbon materials. Though, PPCs have shown distinct differences in structures compared with PP according to their IR results. More detailed information on conjugated structures in PPCs can be obtained by the extraction of several key fitted peaks, which have been deconvoluted from the pristine broad peaks in Raman spectra of PPCs as shown in Figure 5. The fundamental vibration of the *E*_2g_ stretching modes of all pairs of *sp^2^* carbon atoms in aromatic rings (G band) and symmetry breaking at the edges of graphite planes in *sp^2^* carbon (D band) have been well accepted in graphite Raman research [17,18,19]. Different from the highly ordered conjugated structure of graphite, PPCs have broad Raman peaks including their neighboring carbonaceous satellite peaks associated with other functional groups in PPCs. 

To be specific, these broad Raman peaks can be deconvoluted into several pseudo-subpeaks [20,21,22], which are summarized in Table S1. The peak *I*_O_ at around 1695 cm^−1^ is assigned to the carbonyl C=O structure which is consistent with the corresponding adsorption band in the IR spectrum. The peak *I*_G_ at 1590 cm^−1^ represents aromatic ring quadrant breathing rather than *E*_2g_ fundamental vibration for graphite in PPCs with consideration of no convincing signs for the formation of graphitic crystallite structures under low-temperature thermal treatment. Both peaks *I*_G*_ at 1516 cm^−1^ and *I*_C_ at 1432 cm^−1^ represent aromatic semicircle ring stretch for aromatic ring systems with more than two fused benzene rings in the amorphous carbon structure. The peak *I*_D_ at 1350 cm^−1^ is assigned to *sp^2^* carbon in aromatics with six or more fused benzene rings but less than that in graphite, which indicates the presence of medium-to-large-sized aromatic rings in PPCs. Moreover, the peak *I*_S_ at 1270 cm^−1^ is assigned to the *sp*^3^ carbon in aromatic structures including alkyl-aryl ether and C–C on hydroaromatic rings derived from lignin in PPCs. The small peak *I*_I_ at 1140 cm^−1^ is assigned to C–H on aromatic rings. Based on these reasonable deductions, the *I*_D_/*I*_G_ ratios of PPCs can be easily calculated. It varies in a range from 0.481 (PPC-150) to 0.622 (PPC-350), which indicates the thermal treatment temperature plays a key role in the formation of highly conjugated structures in PPCs.

The XPS analysis was performed in order to verify the chemical changes of PPCs under different thermal treatment temperatures. Based on the wide-scan XPS spectra results (Figure S3), both PP and PPCs have the *C-1s*, *N-1s*, *O-1s*, *K-3p*, and *Ca-3p* peaks but extra *P-2s* and *P-2p* peaks are only observed in the PPCs’ XPS spectra. 

**Figure 6:**
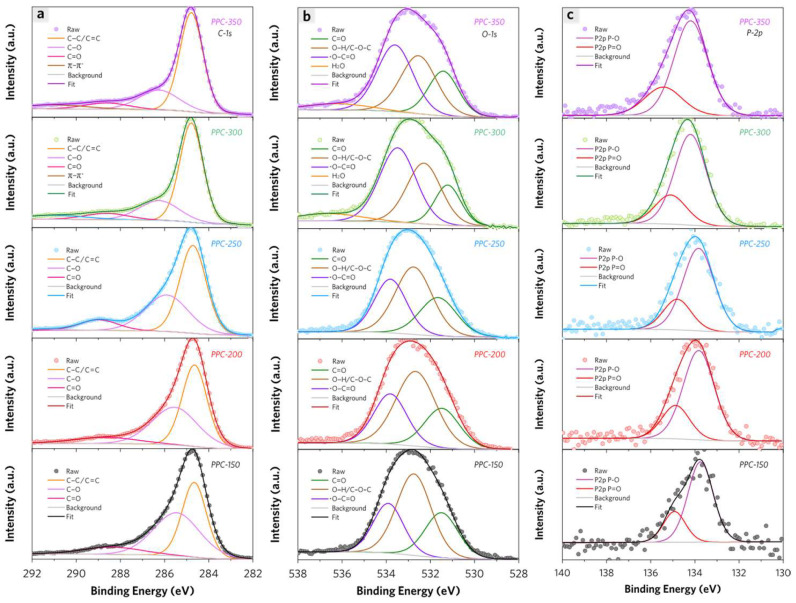
High-resolution XPS patterns of PPCs including (**a**) C-1s, (**b**) O-1s, (**c**) P-2p spectra of PPCs with deconvoluted curves in detail.

As shown in Figure 6a, the *C-1s* high-resolution spectra of PPCs have been performed deconvolution of overlapped peaks: the peak at 284.6 ± 0.2 eV which is assigned to both C–C and C=C bonds, the peak at 285.9 ± 0.2 eV which is assigned to C–O bonds in alcohol and ether groups, the peak at 288.8 ± 0.2 eV which is attributed to C=O bonds of ester and a carboxylic acid, and the satellite peak at 291.0 eV which is attributed to the *C-1s* shake-up effect due to π–π^*^ transition of as formed enhanced aromatic systems in PPCs [6]. The increase of the C–C, C=C peak area indicates the enhanced π-conjugated structure of PPCs through thermal treatment. The decrease of the C–O peak area reveals the decomposition of cellulose in PPCs as the thermal treatment temperature rises, which is consistent with the thermal analysis results. 

In Figure 6b, the deconvolution peaks are achieved from *O-1s* high-resolution spectra of PPCs. Except for the peak ascribed as O sites in adsorbed water, the peaks at nearly 531.5 eV (peak 1), 532.6 eV (peak 2), and 533.6 eV (peak 3) are successively assigned to O sites in C=O, O–H/C–O–C and O*–C=O bonds. The specific integrated area of peak 2 relative to peak 3 changes from 2.00 (PPC-150) to 0.77 (PPC-300), revealing that the enhanced decomposition of cellulose as thermal treatment temperature rises from 150 °C to 300 °C. On the other hand, the hydroxyl groups are considered the most probable adsorption sites in PPC-150, PPC-200, and PPC-250. PPC-300 has more content of carboxyl groups as adsorption sites. As for PPC-350, this specific value is only 0.1 more than that of PPC-300, which is likely due to the enhanced decarboxylation process during thermal treatment above 300 °C. 

According to the deconvolution of the overlapped peak for *P-2p* high-resolution spectra, the peak at 133.9 eV is ascribed as P–O, and the peak at 134.9 eV is ascribed as P=O (Figure 6c). The phosphate ester with terminal hydroxyl anionic groups (adsorbent–O–PO(O^–^)_2_) are also probable adsorption sites for MB molecules. The content of these sites has no significant change among PPCs [10].

### 2.4. Adsorption Isotherms and Thermodynamics

**Figure 7:**
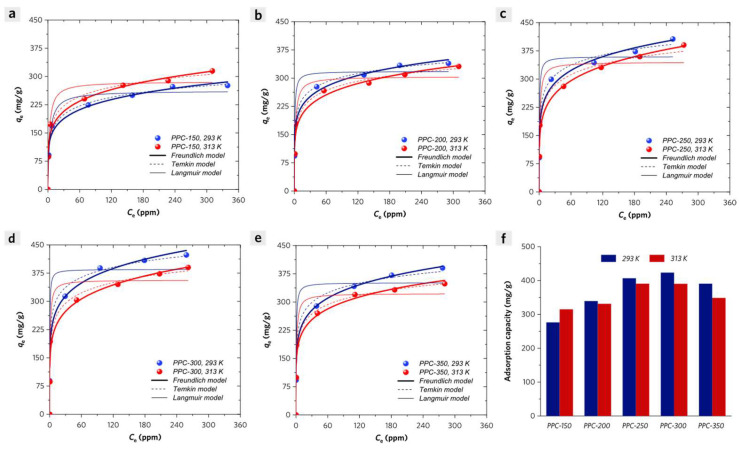
Adsorption isotherms for methylene blue (MB) on PPC-150 (**a**), PPC-200 (**b**), PPC-250 (**c**), PPC-300 (**d**), and PPC-350 (**e**) at 293 K and 313 K; (**f**) The adsorption capacity of PPCs with an initial MB concentration of 500 ppm, an adsorbent dosage of 0.5 g/L at 293 K and 313 K.

The adsorption isotherms of PPCs can be classified as an H-type isotherm shape, which reveals that PPCs exhibit strong affinity toward MB molecules even under low initial concentration region (Figure 7a–e). Three isotherm models (Langmuir, Freundlich, and Temkin equations) are used to simulate the experimental data for understanding the adsorption mechanism of MB on PPCs [23,24].

#### 2.4.1. Langmuir Isotherm Model

Adsorbates are adsorbed by specific homogeneous sites within the adsorbent in monolayer adsorption type, which can be described by the Langmuir isotherm equation (Equation (1)):

(1) $$q_{e}=\frac{Q_{\max}\cdot K_{L}\cdot c_{e}}{1+K_{L}\cdot c_{e}}$$

where *q_e_* is the equilibrium solid phase concentration of adsorbates (mg/g), *c_e_* is the equilibrium liquid phase concentration of adsorbates (mg/L), *Q*_max_ and *K_L_* are the Langmuir parameters related to the theoretical maximum monolayer adsorption capacity and adsorption energy, respectively. However, the experimental adsorption equilibrium data exhibits a monotonic increase in their adsorption equilibrium capacity without an upper limit, which seems to be different from the Langmuir model. Nevertheless, it is correlated with the Langmuir model with adjusted *R*^2^ ranging from 0.923 to 0.977 (Table 2). As a result, the maximum adsorption capacities of PPCs to MB have been exhibited in the following order: PPC-300 > PPC-350, PPC-250 > PPC-200 > PPC-150. As the temperature rises from 293 K to 313 K, a decrease in the *Q*_max_ for MB adsorption on PPCs can be observed except for PPC-150.

#### 2.4.2. Freundlich Isotherm

Heterogeneous surface sites with a nonuniform distribution of adsorption energy within the adsorbent are prevailing according to the basic assumption of Freundlich isotherm presented as Equation (2):

(2) qe=KL·ce1n

where *q_e_* is the equilibrium solid phase concentration of adsorbates (mg/g), *c_e_* is the equilibrium liquid phase concentration of adsorbates (mg/L), *K_L_* and *n* are Freundlich isotherm parameters related to adsorption capacity and intensity, respectively. PPCs have variable *n* values ranging from 5.60 to 6.90 (Henry region: 1 < *n* < 10) with an adjusted *R*-square value range of 0.919–0.971, which indicates a favorable isotherm shape (Table 3).

#### 2.4.3. Temkin Isotherm

The Temkin model is on the basis of two major assumptions: (1) uniform distribution of heterogeneous binding sites on the solid adsorbent surface and (2) linear correlation of binding energy over these different binding sites. It is expressed by the following Equation (3):

(3) $$\frac{q_{e}}{q_{m}}=\frac{RT}{b_{t}}\cdot \ln\left(K_{t}\cdot c_{e}\right)$$

where *q_e_* and *q_m_* is equilibrium and maximum solid phase concentration of adsorbates (mg/g), *c_e_* is the equilibrium liquid phase concentration of adsorbates (mg/L), *K_t_* is the Temkin constant related to adsorption energy, *b_t_* is related to the heat of adsorption (J/mol), *R* is the gas constant (8.314 J mol^–1^ K^–1^) and *T* is the absolute temperature (K).

Here, Equation (4) can be recast as a two-parameter expression with *Q* term standing for *q*_m_ *RT* / *b*_t_ as follows:

(4) $$q_{e}=Q\cdot \ln\left(K_{t}\cdot c_{e}\right)$$

As *q_m_* is unable to be estimated by the Temkin model, the equilibrium adsorption capacity of PPCs to MB with an initial MB concentration of 500 ppm will be used to calculate *b_t_* (heat of adsorption) [25]. PPC-150 exhibits an increase of adsorption energy from 20.2 kJ/mol (20 °C) to 22.7 kJ/mol (40 °C), indicating that high temperature is favorable to the adsorption of MB. Except for PPC-150, PPCs have a decrease in adsorption energy as the temperature rises from 293 K to 313 K (Table 4).

### 2.5. Adsorption Thermodynamics

Thermodynamics is favorable for predicting adsorption mechanisms, the standard change of Gibbs free energy in the adsorption process can be calculated as below:

(5) $$\Delta G^{o}=-RT\ln K_{c}$$

where *R* is the universal gas constant, *T* is the temperature in Kelvin, *K_c_* is a standard equilibrium constant. *K_c_* can be obtained as a dimensionless parameter by multiplying *K*_L_ (Langmuir equilibrium constant) by the molecule weight of adsorbate (MB, 373.90 g/mol), 1000 mg/g, and then *C*_ref_ (0.1363 mol/L for the saturated concentration of MB) according to the kinds of literature [26,27,28].

Standard enthalpy (*∆H^o^*) and entropy (*∆S^o^*) of the adsorption of MB on PPCs can be estimated from Van’t Hoff equation given as follows:

(6) $$\ln K_{c}=\frac{-\Delta H^{o}}{RT}+\frac{\Delta S^{o}}{R}$$

The slope and intercept (ln*K*_c_ versus 1/*T*) are equal to –∆*H^o^*/*R* and ∆*S^o^*/*R*, respectively. The estimated adsorption thermodynamics parameters have been listed in Table 5. Apart from other PPCs, PPC-150 exhibits a positive adsorption enthalpy energy variation of 12.3 kJ/mol, which indicates its endothermic adsorption process. With the existence of hemicellulose and cellulose in PPC-150, the formation of dipole-dipole H-bonding (N→H–O–adsorbent) and Yoshida H-bonding with electron transfer from aromatic rings (framework structure of MB) to hydroxyl groups (PPC-150) are probably major adsorption mechanisms [29,30,31].

### 2.6. Adsorption Kinetics

#### 2.6.1. Adsorption Empirical and Reaction Models

In this study, we applied the adsorption empirical model (Elovich model), pseudo-first-order (PFO) model, and pseudo-second-order (PSO) model to describe the adsorption kinetic process of methylene blue (MB) on PPCs. The model equation parameters and the adjusted correlation coefficients (adj. *R*^2^) values are calculated by the non-linear fitting method [32].

On the basis of two fundamental assumptions: (1) the active energy increases as contact time goes on and (2) the heterogeneous surface properties of adsorbent, the Elovich equation has been commonly used to model a lot of adsorption processes. As an empirical model, it delivered no definite physical meaning. The Elovich model has been described by Equation (7) as follows:

(7) $$q_{t}=\frac{1}{\beta }\cdot \ln\left(1+\alpha \beta t\right)$$

where *q_t_* (mg/g) represents the amount of adsorbate on the adsorbent at any contact time *t* (min), *α* is the initial apparent adsorption rate (mg g^−1^ min^−1^), and *β* (g mg^−1^) is related to the desorption constant.

**Figure 8:**
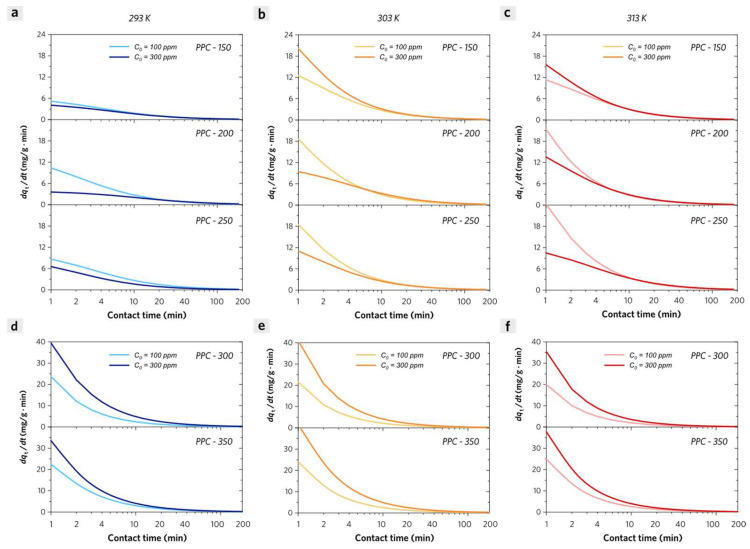
Apparent adsorption rate curves of MB on PPC-I (**a**) at 293 K, (**b**) at 303 K, (**c**) at 313 K and PPC-II (**d**) at 293 K, (**e**) at 303 K, (**f**) at 313 K versus contact time.

The experimental adsorption kinetic data could be well matched by the Elovich equation with high adj. *R*^2^ values (0.998–0.980) in Figure 8. The apparent adsorption rate of MB on PPCs could be calculated according to the following Equation 8:

(8) $$\frac{dq_{t}}{dt}=\frac{\alpha }{1+\alpha \beta t}\;$$

When *t* approaches zero, the *dq_t_*/*dt* is equal to *α* (mg g^−1^ min^−1^), which represents the initial apparent adsorption rate. These values for PPC-300 and PPC-350 (being classified as PPC-II) exhibit much larger than those of PPC-150, PPC-200, and PPC-250 (being classified as PPC-I) in the same experimental conditions. It indicates that there exist very different adsorption kinetic processes for these two groups of PPCs (Table S2). Except for the significant differences in their pore structures, it is evident that the oxygen-containing groups (surface–OH, surface=O, surface–COOH, surface–O–PO(OH)_2_) are considered the main adsorption sites of both PPC-I and PPC-II for dye adsorption. Therefore, we will discuss the MB adsorption kinetic process of PPC-I and PPC-II, separately.

(1) PPC-I. We evaluated the effect of different initial MB concentrations on the adsorption kinetic process with the adsorbent dosage of 0.5 g/L. At a low initial MB concentration (100 ppm), PPC-150 shows a lower apparent adsorption rate than either PPC-200 or PPC-250 at any contact time in Figure 8a–c. In this case, the total quantities of adsorbate (MB) molecules are incapable of achieving saturation adsorption on adsorbent (PPCs), and the desorption of MB from PPCs is negligible. The major adsorption affinity of PPCs (PPC-I) for MB is attributed to the H-bonding (especially for PPC-150) and *n*–π interaction between hydroxyl groups (PPCs) and aromatic fused ring structures (MB). The increased specific surface area is due to the degradation of cellulose and hemicellulose, leading to a major loss of hydroxyl groups and the formation of phosphate ester groups in PPC-200 and especially in PPC-250. The coulombic attraction interactions between cationic groups (tertiary amine groups in MB molecules) and anionic sites groups (carboxyl groups and phosphate ester groups in PPCs) are responsible for the enhanced initial adsorption rate, especially for PPC-200 and PPC-250 at the very beginning of adsorption process. At a high initial MB concentration (300 ppm), PPC-150 shows a higher apparent adsorption rate than PPC-200 and 250 at any contact time. The total quantities of MB molecules are much larger than the maximum MB adsorption capacities of PPCs and the intermolecular interactions between the adsorbed MB molecules (adsorbate on external solid surface) and the free MB molecules (adsorbate in liquid phase) should be addressed. Due to the enhanced pyrolysis in the synthesis of PPC-200 and PPC-250, their adsorption sites of hydroxyl groups are much less than that of PPC-150, which may reduce the major adsorption affinity with a significant decrease in their apparent adsorption rate.

(2) PPC-II. Although there exist intermolecular interactions between the adsorbed MB molecules (on the external solid surface) and the free MB molecules (in the liquid phase), the calculated apparent adsorption rate at a high initial MB concentration (300 ppm) is much higher than that at a low initial MB concentration (100 ppm) in Figure 8d–f. It can be concluded that the adsorbed MB molecules are probably anchored on the internal solid surface of stacked nanopores of PPC-II due to their ultrahigh specific surface areas and porous structures, dramatically blocking the intermolecular interactions due to the limited pore volume for adsorbed MB molecules. The pore-filling effect is supposed to be one of the major effects on the adsorption process of PPC-II and it will be further discussed in the diffusion model section.

The reaction models include the pseudo-first-order (PFO) model and pseudo-second-order (PSO) model, which have been widely adopted to describe the adsorption kinetic processes of adsorbents. Their non-linear-form model equations were presented as Equation (9) (PFO) and Equation (10) (PSO):

(9) $$q_{t}=q_{e}-q_{e}\cdot e^{-k_{1}\cdot t}$$

(10) $$q_{t}=\frac{q_{e}^{2}k_{2}t}{1+q_{e}k_{2}t}$$

where *q_e_*_, *cal*_ (mg/g) and *q_t_* (mg/g) are the amounts of adsorbates on adsorbent at equilibrium and at any contact time *t* (min); *k*_1_ (min^−1^) and *k*_2_ (g mg^−1^ min^−1^) are the adsorption rate constants of the PFO model and PSO model, respectively.

**Figure 9:**
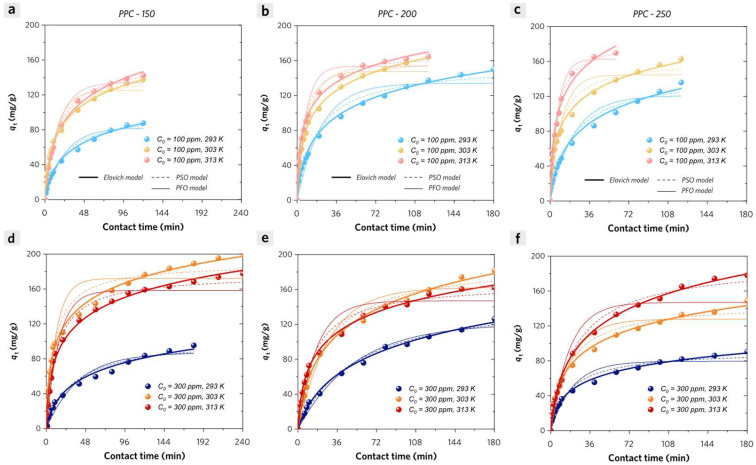
Experimental adsorption kinetic data of methylene blue (MB) on (**a**,**d**) PPC-150, (**b**,**e**) PPC-200, and (**c**,**f**) PPC-250 with different initial concentrations (100 ppm and 300 ppm) of MB at 293 K, 303 K, and 313 K. The fitted Elovich model curves (straight heavy line), pseudo-first-order model curves (straight fine line), and pseudo-second-order model curves (dashed fine line) are also presented.

The fitting results are shown in Figure 9 and the corresponding parameters are also deduced and summarized in Table S3 by using the non-linear fitting method. The experimental kinetic data could be well matched with the PSO model with higher adj. *R*^2^ values as compared with the PFO model. In general, PPC-II adsorbents exhibit much higher adsorption rate constants (*k*_2_) than PPC-I at either low (100 ppm) or high (300 ppm) initial MB concentration. To be specific, the lower initial MB concentration and the higher temperature prefer a larger initial-stage adsorption rate constant for MB adsorption on PPCs. Equilibrium adsorption of MB on PPC-II can be attained in a much shorter time (~180 min) at 313 K, because the *q_e_*_, *cal*_ values are much close to the experimental ones. Except for that, the limited experimental adsorption kinetic measured time is not enough to attain equilibrium adsorption of MB on PPC-I (*q_e_*_, *cal*_ values are much less than the experimental ones).

Considering that PPC-I has much smaller specific surface areas than PPC-II, the pore-filling effect can be negligible in their adsorption process. These factors are suitable for evaluating the effect of adsorption active sites on the adsorption kinetic behaviors of MB on PPC-I. Unlike other PPCs, PPC-150 shows much less decrease in adsorption rate constant when initial MB concentration increases from 100 ppm to 300 ppm. It is likely due to the abundant active sites (–OH) on the surface of PPC-150, which are responsible for strong affinity to MB molecules by H-bonding, which is consistent with its endothermic adsorption of chemisorption property. These results are also consistent with the earlier hypothesis of PPC-I adsorption behaviors that are inferred from the Elovich model discussion.

#### 2.6.2. Adsorption Diffusion Models

Due to the ultrahigh specific surface areas of PPC-II, the pore-filling effect probably plays a key role in dominating the adsorption kinetic process of MB on PPC-II, leading to a large initial apparent adsorption rate according to Elovich model analysis. The adsorption of matter in liquid solution on porous adsorbent generally involves three steps: (1) diffusion of the adsorbate molecules through the boundary layer around the adsorbent particle (external film diffusion); (2) diffusion of adsorbate molecules through pore structure which may be due to pore diffusion or surface diffusion or a combination of both (intra-particle diffusion); (3) adsorption on the internal pore surface (surface reaction). In most cases, the surface reaction step is relatively faster than the diffusion step. In a well-mixed batch reactor with forced stirring, the dye concentration gradient in the liquid film is negligible, which can largely reduce or even eliminate the film mass transfer resistance, so film diffusion can be negligible in these adsorption systems in our work. In order to understand how the intra-particle mass transfer (intra-particle diffusion) influences the adsorption kinetic process, a diffusion model based on Fick’s law is applied with the assumption of a spherical adsorbent particle with an average radius of *R*. The relationship between the dye adsorption quantity (*q_t_*) and contact time (*t*) is given by the following Equation (9):

(11) $$\frac{q_{t}}{q_{\infty }}=1-\frac{6}{\pi^{2}}\sum_{n=1}^{\infty }\frac{1}{n^{2}}\exp⟮-\frac{D_{i}n^{2}\pi^{2}t}{R^{2}}⟯\;$$

where *q*_ꝏ_ (mg/g) represents the equilibrium adsorption quantity in the solid phase of adsorbent at infinite time, *R* is the average radius of the spherical adsorbent particle, *D_i_* (cm^2^/s) is the diffusion coefficient.

If *B* is equal to *D_i_* π^2^/*R*^2^, Equation (11) can be simplified as below:

(12) $$\frac{q_{t}}{q_{\infty }}=1-\frac{6}{\pi^{2}}\sum_{n=1}^{\infty }\frac{1}{n^{2}}\exp\left(-n^{2}Bt\right)$$

For a short time, when *q_t_*/*q*_ꝏ_ < 0.3, Equation (12) can be simplified to yield:

(13) $$\frac{q_{t}}{q_{\infty }}=\frac{6}{\pi^{1.5}}\cdot {\left(Bt\right)}^{0.5}$$

which is usually called the Weber-Morris model plotting *q_t_* versus *t* ^0.5^. If it shows a straight line passing through the origin, which means that the adsorption process is controlled by intra-particle diffusion.

For a moderate time, when *q_t_*/*q*_ꝏ_ < 0.85, Equation (10) can be simplified to yield:

(14) $$\frac{q_{t}}{q_{\infty }}=\frac{6}{\pi^{1.5}}\cdot {\left(Bt\right)}^{0.5}-\frac{3}{\pi^{2}}Bt$$

For a long time, when *q_t_*/*q*_ꝏ_ > 0.85, Equation (10) can be simplified to yield:

(15) $$\frac{q_{t}}{q_{\infty }}=1-\frac{6}{\pi^{2}}\exp\left(-Bt\right)$$

**Figure 10:**
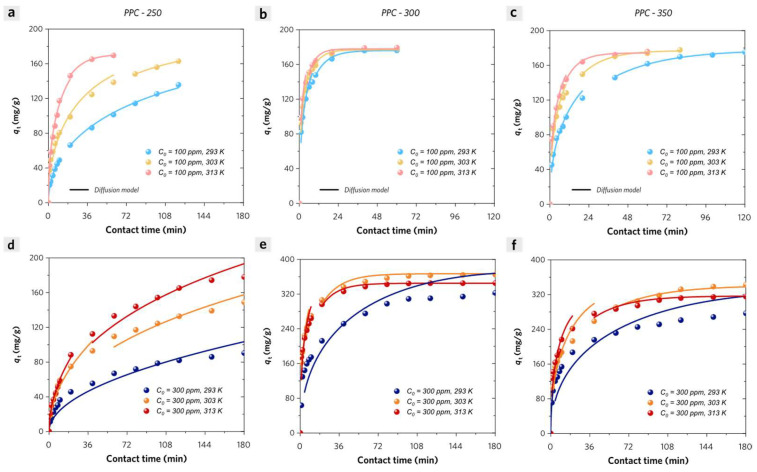
Experimental adsorption kinetic data of methylene blue (MB) on (**a**,**d**) PPC-250, (**b**,**e**) PPC-300, and (**c**,**f**) PPC-350 with different initial MB concentrations (100 ppm and 300 ppm) at 293 K, 303 K, and 313 K. The piece-wise fitted intra-particle diffusion model curves (straight line) are also presented.

Equations (14) and (15) are known as the Boyd or Reichenberg model. According to Figure 10, the experimental adsorption kinetic data can be theoretically simulated by those above piece-wise intra-particle diffusion Equations (13)–(15). The calculated parameters are listed in Table S4. PPC-200 is considered an ideal reference for no intra-particle diffusion contribution to its adsorption kinetic process. At low initial MB concentration (100 ppm), both PPC-I (PPC-200 and PPC-250) and PPC-II (PPC-300 and PPC-350) can be well matched by diffusion models with high adj. *R*^2^ values in most cases. However, the estimated *B* values for PPC-II (0.0312–0.335) are much larger than that for PPC-I (0.0072–0.0592) in the same condition. To be specific, PPC-250 exhibits no intra-particle diffusion contribution to the MB adsorption kinetic process at low temperatures (20–30 °C) except that the estimated *B* value for PPC-250 is above 0.04776 at 40 °C, which is larger than the reference value (0.0376) for PPC-200. It is most likely attributed to the hindered diffusion by its narrow stacked nanopores on the surface of the adsorbent at low temperatures (20–30 °C), whereas the nanopores tend to become open and the adsorbate molecules’ intra-particle diffusion becomes significant at higher temperature. As for PPC-II, because the adsorption of MB is synergistically driven by surface sites and the intra-particle diffusion process, the apparent adsorption rates of PPC-II by the Elovich model can be much higher than that of PPC-I.

At a high initial MB concentration (300 ppm), the experimental kinetic adsorption data of PPC-I and PPC-II cannot be finely matched by those diffusion models except for the final stage of MB adsorption on PPC-II at high temperatures above 30 °C. It is observed that PPC-300 exhibits a much lower estimated B value (0.0146 with adj. *R*^2^ value of 0.73) at high initial MB concentration than that value (0.176 with adj. *R*^2^ value of 0.88) at low initial MB concentration at 20 °C (which is almost the same as the situation of PPC-350), indicating less intra-particle diffusion contributions to apparent adsorption rates of PPC-II at a high initial concentration in this case. While at higher temperatures, the intra-particle diffusion tends to make more contributions to apparent adsorption rates of PPC-II, especially at the final stage of adsorption.

**Figure 11:**
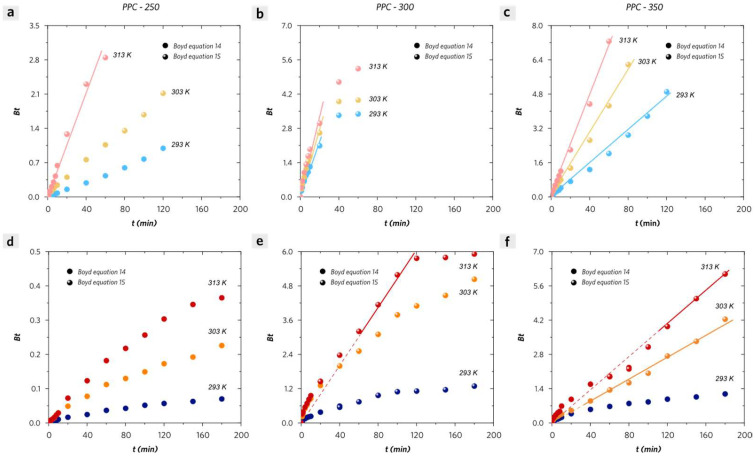
Boyd plots of MB adsorption on (**a**,**d**) PPC-250, (**b**,**e**) PPC-300, and (**c**,**f**) PPC-350 with different initial MB concentrations (**a**–**c**) 100 ppm and (**d**–**f**) 300 ppm at 293 K, 303 K, and 313 K.

In order to understand how the intra-particle diffusion contributes to the kinetic adsorption process of PPCs, the plots of *Bt* versus *t* are illustrated in Figure 11, which is called the Boyd plot. It means that the adsorption process is controlled by intra-particle diffusion if it shows a straight line passing through the origin [33]. According to Figure 11a,d, PPC-250 exhibits less intra-particle-diffusion-controlled adsorption kinetic process due to its low estimated B values at either low or high initial MB concentrations. Abundant surface sites on PPC-250 which mainly includes hydroxyl groups (–OH), phosphate ester groups (–O–P(O)(OH)_2_), and carboxyl groups (–COOH), make contributions to the adsorption affinity driven by intermolecular forces between adsorbents and adsorbates such as coulombic attraction forces, H-bonding, n–π and π–π interactions. PPC-300 and PPC-350 exhibit more intra-particle-diffusion-controlled adsorption kinetic processes at low initial MB concentration (Figure 11b,c). Whereas PPC-300 and PPC-350 no longer followed the intra-particle-diffusion-controlled principle at high initial MB concentration at low temperature (20 °C). Although at high temperatures, the plots show the straight line passing through the origin only at the very first and final stage of the adsorption process according to Figure 11e,f. These results are consistent with the hypothesis concluded by the Elovich model discussion and pore-filling experimental results depicted in Supporting Information (Figure S4). It is concluded that the steric hindrance effect has a major influence on decreasing the intra-particle diffusion coefficient at the middle stage of the adsorption process for PPC-II. In addition, the extra desorption experimental results have also been discussed to comprehensively understand the adsorption mechanisms (Figures S5–S8).

## 3. Materials and Methods

### 3.1. Materials and Reagents

The chemical reagents including phosphoric acid (H_3_PO_4_, AR, ~85%), sodium hydrate (NaOH, AR, ~99%), and methylene blue (MB) were purchased from Sinopharm Chemical Reagent Co., Ltd (Shanghai, China). and local chemical reagent companies in Tianjin. Red-pulp pomelos were purchased from a local market and their peels were sundried and collected for further use (denoted as PP). The deionized water was produced in our lab. 

### 3.2. Preparation of Pomelo Peel Biochar

PPCs were synthesized by modified steps reported in our previous work [10]. PPC-150, PPC-200, PPC-250, PPC-300, and PPC-350 were obtained eventually at different specific pyrolysis temperatures ranging from 150 °C to 350 °C. The detailed synthesis methods are mentioned in the Supporting Information.

### 3.3. Batch Adsorption Experiments

Batch adsorption experiments were conducted in centrifuge tubes with 10 mg PPC in 20 mL dye solution at different initial concentrations. Adsorption isotherms were plotted by measuring the equilibrium MB concentration in the aqueous solution and calculating the saturated adsorption capacity of MB (adsorbate) on PPCs (adsorbent). The amount of MB adsorbed on PPC was calculated from the mass balance equation as follows:

$$q_{e}=\frac{c_{0}-c_{e}}{M}\cdot V$$

where *c*_0_ and *c_e_* are the initial and equilibrium dye concentrations (mg/L), *q_e_* is the equilibrium adsorption capacity of adsorbate (mg/g), while *M* and *V* are the weight of the adsorbent (g) and the volume of dye solution (L), respectively.

Batch kinetic experiments were conducted by mixing 50 mg PPC with 100 mL MB solution with a specific initial concentration in a specially-made double-layer reactor (see Figure S1 in Supporting Information). The adsorption kinetic curves (adsorption quantities of MB on PPCs versus contact time) with different initial MB concentrations and specific temperatures were recorded.

## 4. Conclusions

In summary, we prepared a series of PPC adsorbents with specific change laws of the number of surface sites and porous structures: (i) the densities of hydroxyl groups as surface sites of PPCs are ranked as follows: PPC-150 > PPC-200 > PPC-250 > PPC-300 > PPC-350; (ii) the densities of phosphate ester groups as surface sites of PPCs are ranked as follows: PPC-350 > PPC-300 > PPC-250 > PPC-200 > PPC-150; (iii) the specific surface area of PPCs are ranked as follows: PPC-300 (1274.58 m^2^/g) > PPC-350 (1094.91 m^2^/g) > PPC-250 (511.38 m^2^/g) > PPC-200 (29.46 m^2^/g) > PPC-150 (3.07 m^2^/g); (iv) the pore volumes and average mesopore diameters of PPCs are ranked as follows: PPC-350 (1.30 cm^3^/g, 5.73 nm) > PPC-300 (0.93 cm^3^/g, 4.10 nm) > PPC-250 (0.27 cm^3^/g, 4.00 nm). PPC-300 exhibits the highest adsorption capacity of MB (423 mg/g) at 20 °C with an initial MB concentration of 500 ppm and an adsorbent dosage of 0.5 g/L. After considering the factors discussed in the above sections, the probable adsorption mechanisms of PPCs for MB sorption are concluded as below:

(1) The adsorption of MB on PPC-150 mainly exhibits the character of chemisorption with positive enthalpy energy of adsorption (12.3 kJ/mol), which is likely due to the formation of dipole-dipole H-bonding (N→H–O–adsorbent) and Yoshida H-bonding with electron transfer from aromatic rings (framework structure of MB) to hydroxyl groups (major surface sites of PPC-150);
(2) The adsorption of MB on PPCs except for PPC-150 mainly exhibits characters of physisorption with negative enthalpy energy of adsorption (–25.0 to –32.8 kJ/mol). Desorption becomes significant at high temperatures (above 30 °C), leading to the decrease of equilibrium adsorption quantities of MB on PPCs;
(3) PPC-300 and PPC-350 exhibit much higher apparent adsorption rates than either PPC-250 or PPC-200, leading to a rapid adsorption equilibrium due to synergetic contributions of surface adsorption (active sites) and pore-filling (intra-particle diffusion);
(4) Especially, PPC-300 and PPC-350 exhibit intra-particle-diffusion-controlled adsorption kinetic process with low initial MB concentration (100 ppm) or at the very beginning and final stage of adsorption with high initial MB concentration (300 ppm) at 40 °C.

## Figures and Tables

**Table 1:** Textural and pore structural parameters of PP and PPCs.

Sample Name	S_BET_ (m^2^/g)	S_meso_ (m^2^/g)	V_total_ (cm^3^/g)	V_micro_ (cm^3^/g)	V_meso_ (cm^3^/g)	Mid-Value D_micro_ (nm)	Average D_meso_ (nm)
***PP***	0.5503	–	0.00143	–	–	–	–
***PPC-150***	3.065	–	0.015	–	–	–	–
***PPC-200***	29.463	25.854	0.039	–	0.038	–	–
***PPC-250***	511.380	101.325	0.267	0.2017	0.101	0.6005	3.994
***PPC-300***	1274.577	646.040	0.927	0.5105	0.661	0.6444	4.095
***PPC-350***	1094.909	824.592	1.298	0.4559	1.181	0.6726	5.728

**Table 2:** Langmuir isotherm parameters of the adsorption process of MB on PPCs.

Isotherm Model	Temperature	Parameters	Samples				
			*PPC-150*	*PPC-200*	*PPC-250*	*PPC-300*	*PPC-350*
***Langmuir***	293 K	*Q*_max_	262.08	318.05	359.72	385.23	351.40
		*K*_L_	0.271	1.360	1.783	2.750	1.797
		*Adj. R* ^2^	0.974	0.968	0.955	0.946	0.936
	313 K	*Q*_max_	286.57	304.30	345.06	356.90	322.68
		*K*_L_	0.374	0.545	0.829	1.162	0.932
		*Adj. R* ^2^	0.967	0.969	0.946	0.961	0.958

**Table 3:** Freundlich isotherm parameters of the adsorption process of MB on PPCs.

Isotherm Model	Temperature	Parameters	Samples				
			*PPC-150*	*PPC-200*	*PPC-250*	*PPC-300*	*PPC-350*
***Freundlich***	293 K	*K*_L_	104.39	153.46	171.12	187.50	163.17
		*n*	5.79	6.90	6.45	6.60	6.37
		*Adj. R* ^2^	0.951	0.919	0.947	0.966	0.935
	313 K	*K*_L_	113.15	131.80	142.33	155.62	149.56
		*n*	5.62	6.19	5.60	6.04	6.47
		*Adj. R* ^2^	0.944	0.926	0.971	0.932	0.922

**Table 4:** Freundlich isotherm parameters of the adsorption process of MB on PPCs.

Isotherm Model	Temperature	Parameters	Samples				
			*PPC-150*	*PPC-200*	*PPC-250*	*PPC-300*	*PPC-350*
***Temkin***	293 K	*Q*	33.29	32.90	38.68	38.72	37.10
		*K_t_*	13.00	110.51	101.26	201.67	104.66
		*b_t_* (kJ/mol)	20.212	25.130	25.613	26.631	25.657
		*Adj. R^2^*	0.992	0.974	0.986	0.992	0.968
	313 K	*Q*	36.11	35.53	40.94	39.80	35.84
		*K_t_*	15.50	30.74	33.76	55.06	59.52
		*b_t_* (kJ/mol)	22.704	24.259	24.821	25.519	25.318
		*Adj. R^2^*	0.985	0.977	0.987	0.974	0.973

**Table 5:** The estimated adsorption thermodynamics parameters of PPCs.

Samples	Parameters			∆*H^o^* (kJ/mol)	∆*S^o^* (J/mol K)
	*T* (K)	*K* *_c_*	∆*G^o^* (kJ/mol)		
***PPC-150***	293	13,811	−23.223	12.281	121.2
	313	19,060	−25.646		
***PPC-200***	293	69,309	−27.152	−34.862	−26.3
	313	27,774	−26.626		
***PPC-250***	293	90,866	−27.812	−29.196	−4.7
	313	42,248	−27.718		
***PPC-300***	293	140,147	−28.868	−32.841	−13.6
	313	59,218	−28.596		
***PPC-350***	293	91,580	−27.831	−25.030	9.6
	313	47,497	−28.022		

## Data Availability

The data presented in this study are available on request from the corresponding author.

## Supplementary Materials

The following supporting information can be downloaded at: https://www.mdpi.com/article/10.3390/molecules28114426/s1, Figure S1: schematic of continuous kinetic adsorption experimental reactor; Figure S2: FTIR spectrum of PP; Figure S3: wide-scan XPS spectra of PP and PPCs; Figure S4: specific surface areas and pore size distribution bar charts of PPCs: (a) PPC-250, (d) PPC-300, and (g) PPC-350; (b) PPC-250, (e) PPC-300, and (h) PPC-350 with adsorbed MB within a contact time of 20 min, with *c*_0_ = 100 ppm, an adsorbent dosage of 0.5 g/L, at 30 °C; (c) PPC-250, (f) PPC-300, and (i) PPC-350 with adsorbed MB within a contact time of 360 min, with *c*_0_ = 300 ppm, an adsorbent dosage of 0.5 g/L, at 30 °C; Figure S5: SEM images and EDS patterns with atomic ratio of C, O, and P element of (a) PPC-150, (b) PPC-200, (c) PPC-250, (d) PPC-300, (e) PPC-350, (f) MB-adsorbed PPC-150, (g) MB-adsorbed PPC-200, (h) MB-adsorbed PPC-250, (i) MB-adsorbed PPC-300, (j) MB-adsorbed PPC-350; Figure S6: Photographs of solutions obtained from MB-adsorbed PPCs in a pure water system (PPC-150 to PPC-350 from left to right) within 24 h desorption experiment; Figure S7: IR spectra of PPCs, MB-adsorbed PPCs after water extraction, and MB-adsorbed PPCs after multiple ethanol elution processes; Figure S8: TEM images of PPC-300 (a,b,c) and MB-adsorbed PPC-300 (d,e,f) at different magnifications; Table S1: summary of the deconvoluted peaks assignment in the Raman spectra of PPCs; Table S2: estimated kinetic parameters of the Elovich model by the non-linear fitting method; Table S3: estimated kinetic parameters of PFO and PSO model by the non-linear fitting method; Table S4: estimated kinetic parameters of intra-particle diffusion model by the non-linear fitting method.

## Sample Availability

Samples of the PPCs are available from the authors.
