# Supplementary material for: Revealing the Combined Effect of Active Sites and Intra-Particle Diffusion on Adsorption Mechanism of Methylene Blue on Activated Red-Pulp Pomelo Peel Biochar
Source: Molecules. 2023 May 29;28(11):4426. doi: 10.3390/molecules28114426 (PMC10254690; doi:10.3390/molecules28114426)
Supplement: Supplementary file 1 [file molecules-28-04426-s001.zip › molecules-2389143-supplementary.pdf]

Supplementary data

Revealing the Combined Effect of Active Sites and Intra-Particle Diffusion on Adsorption Mechanism of Methylene Blue on Activated Red-Pulp Pomelo Peel Biochar

Fang Wei[1], Shenglong Jin[1], Chunyi Yao[1], Tianhao Wang[1], Shengpu Zhu[1], Yabiao Ma[1], Heng Qiao[1], Linxi Shan[1], Rencong Wang[1], Xiaoxue Lian[1], Xiaoqiang Tong[1], Yan Li[1], Qiang Zhao[1,*], Weiguo Song[2]

[1] College of Science, Civil Aviation University of China (CAUC), Tianjin, 300300, China

[2] Laboratory of Molecular Nanostructure and Nanotechnology, Institute of Chemistry, Chinese Academy of Sciences, Beijing, 100190, China

* Corresponding author. College of Science, Civil Ation University of China (CAUC), Tianjin, 300300, China

Email address: zhao-q@cauc.edu.cn (Q. Zhao).

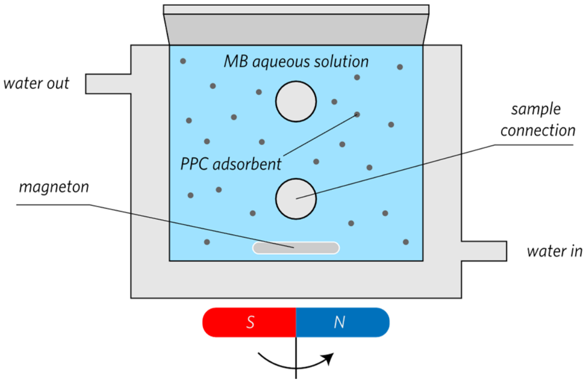

Figure S1. Schematic of continuous kinetic adsorption experimental reactor.

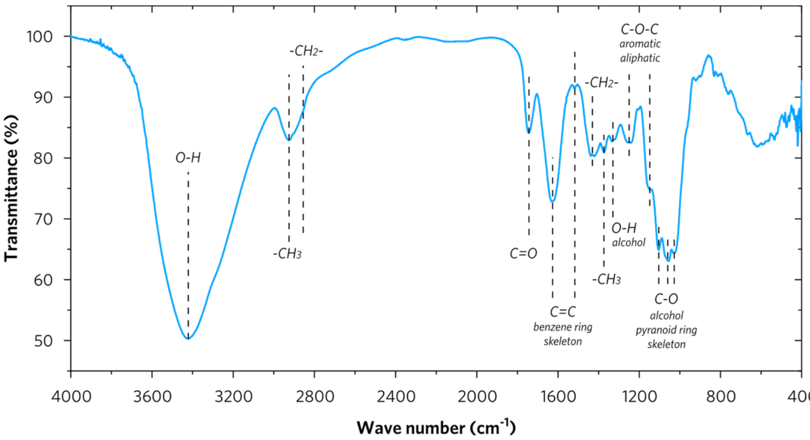

Figure S2. FTIR spectrum of PP.

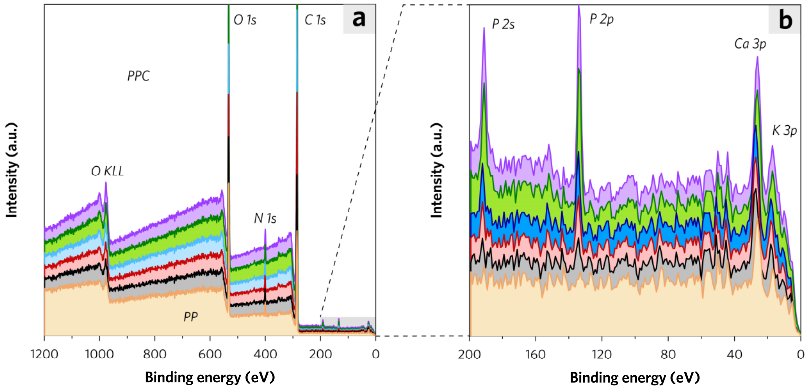

Figure S3. Wide-scan XPS spectra of PP and PPCs.

The pore-filing experimental results of MB adsorption on PPC-250 and PPC-II (PPC-300 and PPC-350) are shown in **Fig. S4**. The specific surface areas (by BET and BJH model) of PPC-250, PPC-300, and PPC-350 at initial adsorption contact time are measured. At a low initial MB concentration (100 ppm), the specific surface areas of PPC adsorbents (separated from MB aqueous solution while contact time is 20 min and then dried) accompanied with their pore size distribution bar charts are also measured as well as the calculated q_t at a contact time of 20 min and 30 ºC. At a high initial MB concentration (300 ppm), the specific surface areas of PPC adsorbents (separated from MB aqueous solution while contact time is 360 min and then dried) accompanied with their pore size distribution bar charts are also measured as well as the calculated q_t at a contact time of 360 min and 30 ºC. We observe that both the specific surface areas and pore volumes of PPC adsorbents decreased significantly as MB adsorption contact time goes longer. We assume that homogeneous adsorption of MB on the PPC surface (including both external surface and internal surface) happens during MB adsorption on PPCs (adsorbed MB molecules are mainly located in mesopores), the calculated quantity of adsorbed MB molecules per unit area (*n*) on PPCs are listed as below:

Sample	***Contact time and initial concentration***		***Contact time and initial concentration***	
	20 min	*100 ppm*	*360 min*	*300 ppm*
	ΔS_{BJH}	***n*** */nm*^{2}	ΔS_{BJH}	***n*** */nm*^{2}
PPC-250	*56.2 m*^{2}*/g*	*3.3*	–	–
PPC-300	*472.5 m*^{2}*/g*	*0.63*	*633.6 m*^{2}*/g*	*1.15*
PPC-350	*415.4 m*^{2}*/g*	*0.63*	*578.9 m*^{2}*/g*	*1.15*

–: Not reach equilibrium adsorption within finite contact time.

Due to its narrow stacked nanopores, adsorbate molecules can hardly diffuse into deep holes during MB adsorption on PPC-250 within 20 min at 30 ºC. The decreased surface area is likely attributed to the massive stack of adsorbate molecules inside the channel adjacent to the external surface of PPC-250. As for PPC-II, the calculated quantity of adsorbed MB molecules per unit area (n) is nearly 0.63 when the contact time is 20 min (c_0 = 100 ppm), which is almost in accordance with the MB molecular size. At nearly equilibrium adsorption of MB on PPC-II, n is estimated to be 1.15, which is smaller than the reciprocal value of the theoretical projected area of MB molecules (1.43). It indicates that the direction of the long axis of the adsorbed MB molecule is most likely parallel to the surface of the PPC adsorbent.

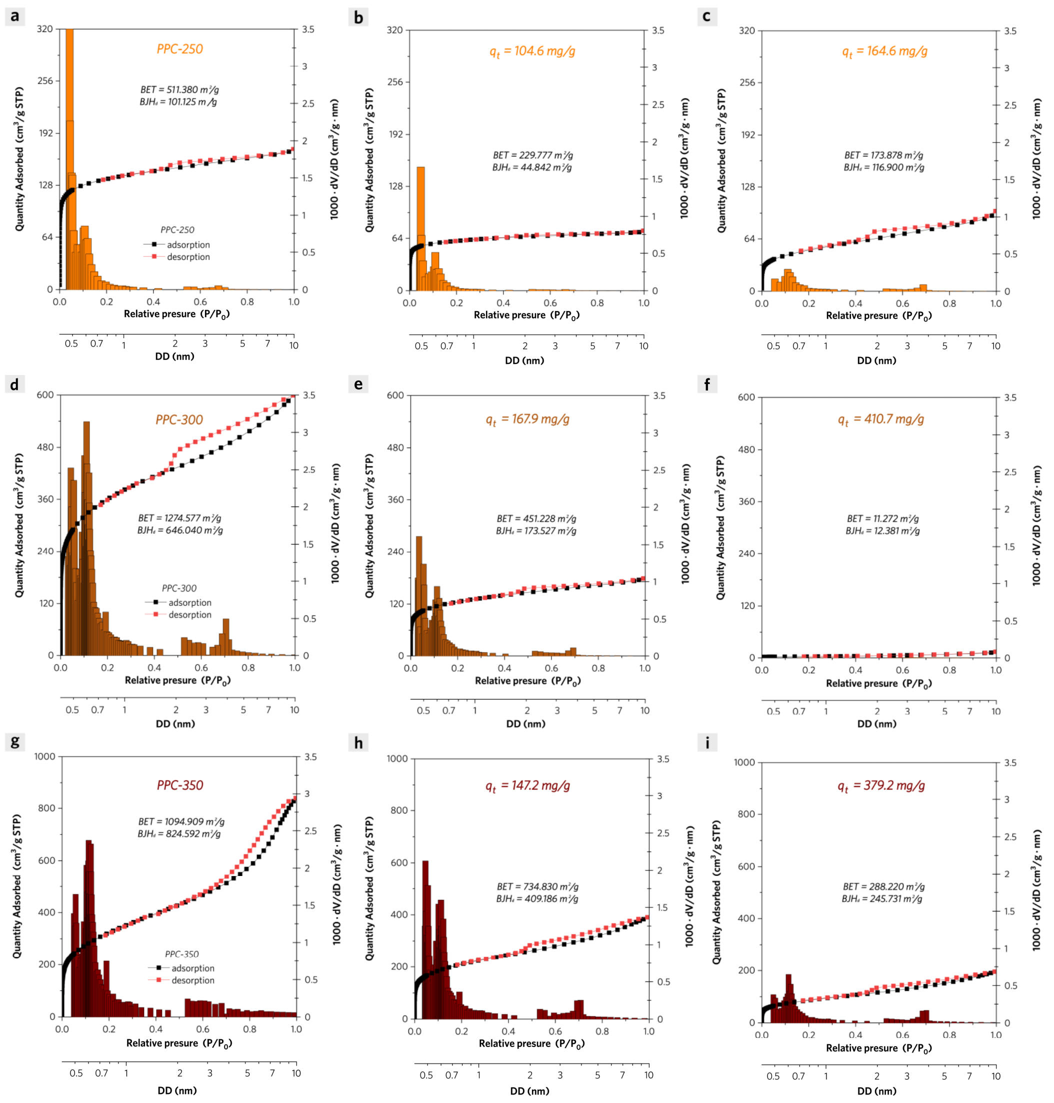

Figure S4. Specific surface areas and pore size distribution bar charts of PPCs: (a) PPC-250, (d) PPC-300, and (g) PPC-350; (b) PPC-250, (e) PPC-300, and (h) PPC-350 with adsorbed MB within a contact time of 20 min, with c_0 = 100 ppm, an adsorbent dosage of 0.5 g/L, at 30 °C; (c) PPC-250, (f) PPC-300, and (i) PPC-350 with adsorbed MB within a contact time of 360 min, with c_0 = 300 ppm, an adsorbent dosage of 0.5 g/L, at 30 °C.

In order to investigate the desorption mechanisms. MB-adsorbed PPCs are prepared within the equilibrium adsorption of MB (300 ppm in 200 mL with an adsorbent dosage of 100 mg for 24 h). The SEM images and their corresponding EDS patterns of PPCs and MB-adsorbed PPCs are shown in **Fig. S5**. We can observe a slight change between pristine PPCs and MB-adsorbed PPCs. MB-adsorbed PPCs have more smooth roughness than PPCs. According to their EDS patterns, there exist specific X-Ray emission peaks of the S element of MB at ~2.32 eV in MB-adsorbed PPCs. It indicates that MB is significantly adsorbed by PPCs.

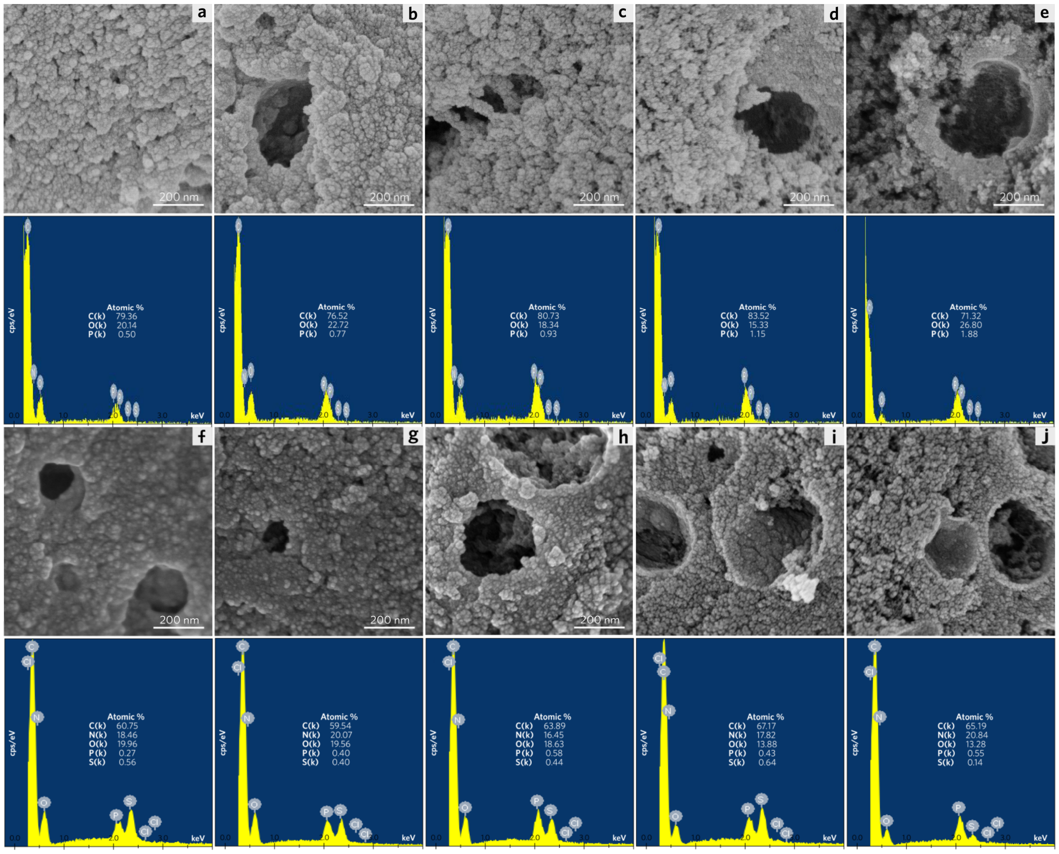

Figure S5. SEM images and EDS patterns with atomic ratio of C, O, and P element of (a) PPC-150, (b) PPC-200, (c) PPC-250, (d) PPC-300, (e) PPC-350, (f) MB-adsorbed PPC-150, (g) MB-adsorbed PPC-200, (h) MB-adsorbed PPC-250, (i) MB-adsorbed PPC-300, (j) MB-adsorbed PPC-350.

Some MB-adsorbed PPCs are dispersed into pure water within 24 h. Finally, the solutions are also obtained as shown in **Fig. S6**. MB-adsorbed PPCs after removing MB have also been prepared with multiple ethanol elution processes. The IR spectra of these solid materials are presented in **Fig. S7**. According to these results, we observe that MB can be partially eluted from MB-adsorbed PPCs by ethanol, but hardly by water. It indicates that ethanol strongly interacts with MB through weak polar interaction whereas water weakly interacts with MB through strong polar interaction (hydrogen bonding). According to the desorption experimental results, PPC-150, PPC-200, PPC-250, and PPC-300 exhibit almost no desorption of MB with water elution. But PPC-350 exhibits a little desorption of MB with water extraction. Because PPC-350 has enhanced conjugated aromatic structure, less density of active sites, and more proportion of micropores, MB can be adsorbed and enriched in stacked micropores through π–π interactions, intermolecular forces, and capillary condensation effects. These adsorbed MB can be partially eluted with water extraction. According to IR results, we can hardly observe the difference between MB-adsorbed PPCs after water extraction and those after ethanol elution. Though MB can be partially eluted from MB-adsorbed PPCs, most of MB molecules are still stable on PPCs, which indicates the interactions between adsorbent and adsorbate are not pure physical adsorption mechanism but complicated mechanisms.

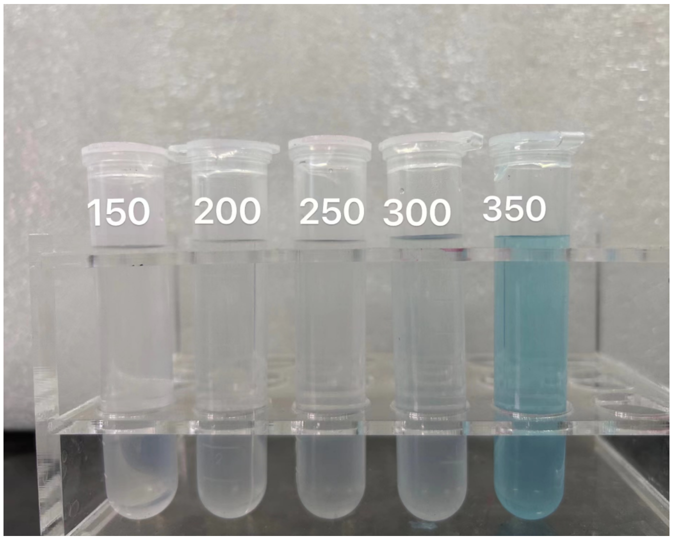

Figure S6. Photographs of solutions obtained from MB-adsorbed PPCs in a pure water system (PPC-150 to PPC-350 from left to right) within 24 h desorption experiments.

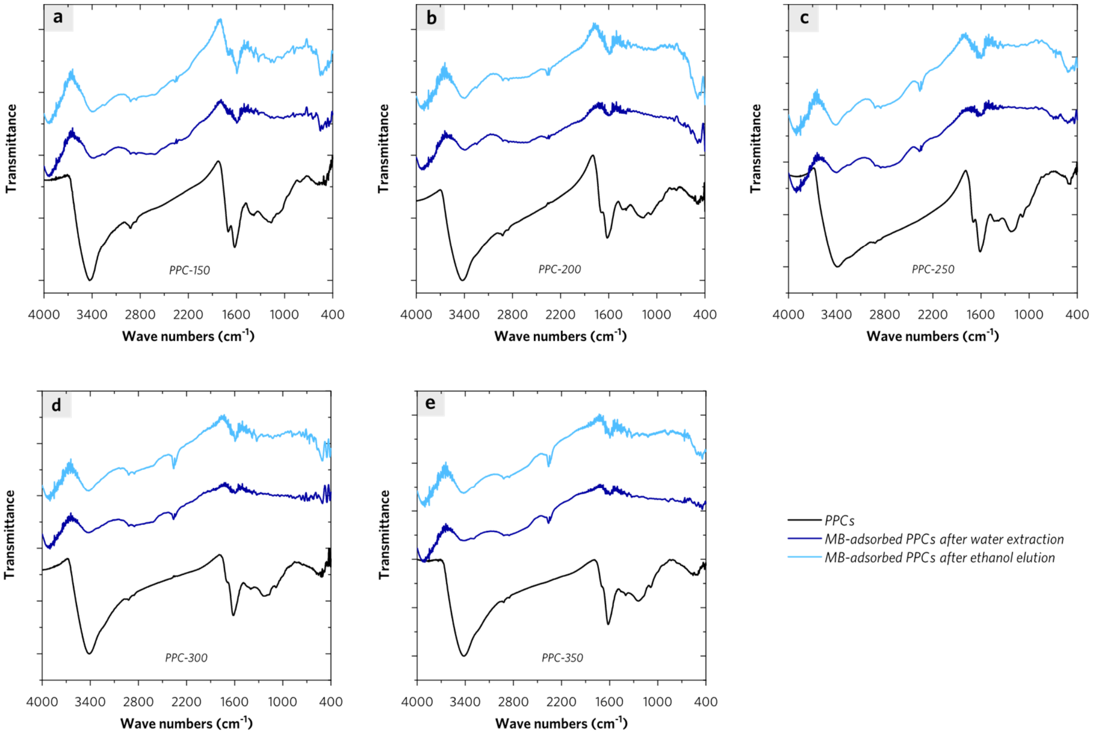

Figure S7. IR spectra of PPCs, MB-adsorbed PPCs after water extraction, and MB-adsorbed PPCs after multiple ethanol elution processes.

TEM images of PPC-300 and MB-adsorbed PPC-300 have been shown in **Fig. S8**. We observe the flexible morphology of pristine PPC-300 with a sign of abundant stacked nanopores. Moreover, the MB-adsorbed PPC-300 also exhibits wide translucent shadows in its bulk structures, which means that there exist significant differences in their surface morphologies between PPC-300 and MB-adsorbed PPC-300.

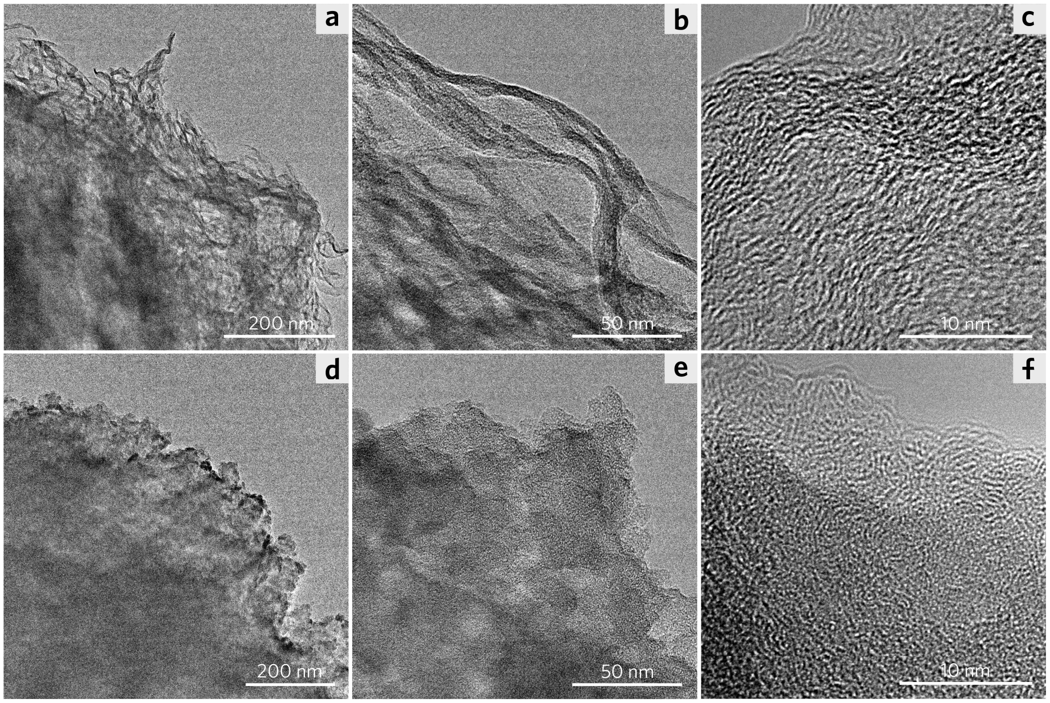

Figure S8. TEM images of PPC-300 (a, b, c) and MB-adsorbed PPC-300 (d, e, f) at different magnifications.

Table S1 Summary of the deconvoluted peaks assignment in the Raman spectra of PPCs.

Peak	*Raman shift*	*Peak assignment*
I_O	1695 cm^{-1}	*carbonyl C=O*
I_G	1590 cm^{-1}	*aromatic ring quadrant breathing*
I_{G*}	1516 cm^{-1}	*aromatic semicircle ring stretches for aromatic ring systems with more than two fused benzene rings in the amorphous carbon structure*
I_C	1432 cm^{-1}	*aromatic semicircle ring stretches for aromatic ring systems with more than two fused benzene rings in the amorphous carbon structure*
I_D	1350 cm^{-1}	*sp^2 carbon in aromatics with six or more fused benzene rings but less than that in graphite*
I_S	1270 cm^{-1}	*sp3 carbon in aromatic structures, especially like alkyl-aryl ether and C–C on hydroaromatic rings in lignin structure*
I_I	1140 cm^{-1}	*C–H on aromatic rings*

No additional instructions.

Table S2 Estimated kinetic parameters of the Elovich model by the non-linear fitting method.

Kinetic model	*Sample*	*Parameters*	*c_0 = 100 ppm*			*c_0 = 300 ppm*		
			293 K	*303 K*	*313 K*	*293 K*	*303 K*	*313 K*
**Elovich**	*PPC-150*	*α*	*6.59*	*21.05*	*16.36*	*5.83*	*50.63*	*29.31*
		β	*0.040*	*0.032*	*0.027*	*0.042*	*0.030*	*0.030*
		Adj. R^2	*0.996*	*0.998*	*0.997*	*0.988*	*0.996*	*0.993*
	PPC-200	*α*	*15.03*	*46.10*	*85.13*	*3.90*	*11.89*	*22.89*
		β	*0.030*	*0.032*	*0.035*	*0.023*	*0.022*	*0.030*
		Adj. R^2	*0.993*	*0.998*	*0.995*	*0.992*	*0.995*	*0.993*
	PPC-250	*α*	*11.62*	*47.29*	*70.45*	*9.88*	*17.54*	*13.66*
		β	*0.029*	*0.033*	*0.027*	*0.051*	*0.032*	*0.022*
		Adj. R^2	*0.986*	*0.992*	*0.989*	*0.996*	*0.995*	*0.995*
	PPC-300	*α*	*823.20*	*2250.09*	*5057.24*	*188.63*	*2501.39*	*4579.76*
		β	*0.041*	*0.046*	*0.050*	*0.020*	*0.024*	*0.028*
		Adj. R^2	*0.989*	*0.981*	*0.980*	*0.991*	*0.990*	*0.988*
	PPC-350	*α*	*79.67*	*378.71*	*464.49*	*173.16*	*247.89*	*688.58*
		β	*0.032*	*0.039*	*0.038*	*0.024*	*0.020*	*0.025*
		Adj. R^2	*0.996*	*0.995*	*0.989*	*0.999*	*0.990*	*0.993*

No additional instructions.

Table S3 Estimated kinetic parameters of PFO and PSO model by the non-linear fitting method.

Kinetic model	*Sample*	*Parameters*	*c_0 = 100 ppm*			*c_0 = 300 ppm*		
			293 K	***303 K***	***313 K***	***293 K***	***303 K***	***313 K***
Pseudo-first-order	*PPC-150*	$q_{e, cal}$	*82.32*	*124.99*	*134.15*	*86.84*	*172.03*	*158.39*
		k_1	*0.043*	*0.071*	*0.060*	*0.026*	*0.086*	*0.066*
		Adj. R^2	*0.964*	*0.951*	*0.982*	*0.943*	*0.896*	*0.957*
	PPC-200	$q_{e, cal}$	*134.08*	*147.59*	*153.76*	*120.91*	*162.29*	*146.96*
		k_1	*0.045*	*0.097*	*0.124*	*0.019*	*0.035*	*0.058*
		Adj. R^2	*0.939*	*0.935*	*0.933*	*0.977*	*0.957*	*0.938*
	PPC-250	$q_{e, cal}$	*120.75*	*144.63*	*162.70*	*79.58*	*127.78*	*163.14*
		k_1	*0.046*	*0.095*	*0.142*	*0.050*	*0.054*	*0.038*
		Adj. R^2	*0.934*	*0.900*	*0.956*	*0.935*	*0.932*	*0.960*
	PPC-300	$q_{e, cal}$	*160.14*	*164.22*	*166.64*	*291.71*	*341.67*	*324.00*
		k_1	*0.446*	*0.583*	*0.695*	*0.122*	*0.273*	*0.324*
		Adj. R^2	*0.917*	*0.938*	*0.952*	*0.890*	*0.878*	*0.848*
	PPC-350	$q_{e, cal}$	*161.76*	*160.93*	*160.84*	*245.87*	*305.58*	*292.11*
		k_1	*0.116*	*0.257*	*0.339*	*0.125*	*0.126*	*0.183*
		Adj. R^2	*0.915*	*0.857*	*0.915*	*0.897*	*0.835*	*0.858*
Pseudo-second-order	*PPC-150*	$q_{e, cal}$	*99.99*	*144.22*	*157.95*	*103.80*	*189.50*	*176.54*
		k_2	*0.00046*	*0.00057*	*0.00042*	*0.00029*	*0.00054*	*0.00045*
		Adj. R^2	*0.985*	*0.983*	*0.996*	*0.969*	*0.956*	*0.989*
	PPC-200	$q_{e, cal}$	*155.45*	*164.94*	*167.69*	*154.56*	*193.26*	*167.38*
		k_2	*0.00034*	*0.00075*	*0.00102*	*0.00012*	*0.00021*	*0.00042*
		Adj. R^2	*0.973*	*0.975*	*0.975*	*0.986*	*0.983*	*0.975*
	PPC-250	$q_{e, cal}$	*144.73*	*161.43*	*184.41*	*91.73*	*146.76*	*192.57*
		k_2	*0.00035*	*0.00075*	*0.00098*	*0.00065*	*0.00043*	*0.00023*
		Adj. R^2	*0.965*	*0.952*	*0.982*	*0.974*	*0.972*	*0.984*
	PPC-300	$q_{e, cal}$	*176.02*	*178.68*	*180.06*	*314.28*	*358.07*	*339.36*
		k_2	*0.00366*	*0.00485*	*0.00584*	*0.00055*	*0.00132*	*0.00167*

	Adj. R^2	*0.983*	*0.990*	*0.994*	*0.953*	*0.964*	*0.950*
PPC-350	$q_{e, cal}$	*177.26*	*173.31*	*176.24*	*264.59*	*328.33*	*307.17*
	k_2	*0.00090*	*0.00239*	*0.00289*	*0.00067*	*0.00056*	*0.00099*
	Adj. R^2	*0.965*	*0.948*	*0.980*	*0.955*	*0.958*	*0.940*

No additional instructions.

Table S4 Estimated kinetic parameters of intra-particle diffusion model by the non-linear fitting method.

Sample	*Kinetic model*	*Parameters*	***c_0 = 100 ppm***			***c_0 = 300 ppm***		
			293 K	***303 K***	***313 K***	***293 K***	***303 K***	***313 K***
PPC-200 *(reference)*	***Weber-Morris*** $q_t/q_\infty < 0.3$	q_∞	*176*	*176*	–	*309*	*298*	*287*
		B	*0.00915*	*0.0282*	–	*0.00082*	*0.00325*	*0.00471*
		Adj. R^2	*0.978*	*0.991*	–	*0.973*	*0.972*	*0.970*
	Boyd $q_t/q_\infty < 0.85$	q_∞	*176*	*176*	*176*	*309*	*298*	*287*
		B	*0.00874*	*0.0239*	*0.0376*	*0.00103*	*0.00307*	*0.00306*
		Adj. R^2	*0.994*	*0.960*	*0.967*	*0.989*	*0.954*	*0.616*
	Boyd $q_t/q_\infty > 0.85$	q_∞	–	–	–	–	–	–
		B	–	–	–	–	–	–
		Adj. R^2	–	–	–	–	–	–
PPC-250	***Weber-Morris*** $q_t/q_\infty < 0.3$	q_∞	*174*	*176*	*177*	*343*	*336*	*330*
		B	*0.0072*	*0.0335*	*0.04776*	*0.00044*	*0.00208*	*0.00287*
		Adj. R^2	*0.985*	*0.997*	*0.999*	*0.931*	*0.956*	*0.991*
	Boyd $q_t/q_\infty < 0.85$	q_∞	*176*	*176*	*177*	–	*336*	*330*
		B	*0.0076*	*0.0219*	*0.0564*	–	*0.00142*	*0.0025*
		Adj. R^2	*0.993*	*0.950*	*0.984*	–	*0.701*	*0.848*
	Boyd $q_t/q_\infty > 0.85$	q_∞	–	*176*	*177*	–	–	–
		B	–	*0.0170*	*0.0592*	–	–	–
		Adj. R^2	–	*0.988*	*0.922*	–	–	–
PPC-300	***Weber-Morris*** $q_t/q_\infty < 0.3$	q_∞	–	–	–	–	–	–
		B	–	–	–	–	–	–
		Adj. R^2	–	–	–	–	–	–
	Boyd $q_t/q_\infty < 0.85$	q_∞	*176*	*176*	*176*	*388*	*367*	*345*
		B	*0.176*	*0.253*	*0.335*	*0.0146*	*0.126*	*0.136*
		Adj. R^2	*0.877*	*0.851*	*0.833*	*0.730*	*0.661*	*0.199*
	Boyd $q_t/q_\infty > 0.85$	q_∞	*176*	*177*	*178*	–	*367*	*345*
		B	*0.131*	*0.187*	*0.221*	–	*0.0579*	*0.070*

		Adj. R^2	*0.988*	*0.968*	*0.974*	–	*0.856*	*0.967*
PPC-350	***Weber-Morris*** $q_t/q_\infty < 0.3$	q_∞	–	–	–	*341*	–	–
		B	–	–	–	*0.0365*	–	–
		Adj. R^2	–	–	–	*0.999*	–	–
	Boyd $q_t/q_\infty < 0.85$	q_∞	*177*	*177*	*174*	*341*	*346*	*314*
		B	*0.0429*	*0.103*	*0.143*	*0.0121*	*0.0378*	*0.0744*
		Adj. R^2	*0.943*	*0.695*	*0.938*	*0.654*	*0.580*	*0.624*
	Boyd $q_t/q_\infty > 0.85$	q_∞	*178*	*177*	*174*	–	*341*	*317*
		B	*0.0312*	*0.0685*	*0.123*	–	*0.0234*	*0.0337*
		Adj. R^2	*0.992*	*0.989*	*0.986*	–	*0.963*	*0.855*

–: No experimental data
